# Circadian clock activity in human umbilical vein endothelial cells of preterm and term neonates

**DOI:** 10.1038/s41390-024-03705-3

**Published:** 2024-12-13

**Authors:** Meilan Zuo, Achim Kramer, Gregor Mönke, Lina K. Sciesielski, Christof Dame

**Affiliations:** 1 Department of Neonatology, Charité - Universitätsmedizin Berlin, corporate Member of Freie Universität Berlin and Humboldt-Universität zu Berlin, Berlin, Germany; 2 Institute of Medical Immunology, Charité - Universitätsmedizin Berlin, corporate Member of Freie Universität Berlin and Humboldt-Universität zu Berlin, Berlin, Germany; 3 Ernst Strüngmann Institute for Neuroscience, Frankfurt/Main, Germany

## Abstract

**Background:**

During mammalian gestation, fetal circadian rhythms are thought to be mainly controlled by maternal signals. In humans, the initiation and activity of central and peripheral circadian clocks is largely unknown. This study aimed to elucidate the developmental clock properties in human umbilical vein endothelial cells (HUVECs).

**Methods:**

HUVECs were obtained from (a) preterm infants, subgrouped according to birth weight or gestational age classification, and (b) term infants (in total: *n* = 60). In vitro clock activity was determined by using live bioluminescence recording of a luciferase reporter gene under circadian control over 120 h. In addition, core clock and clock-associated gene expression were quantified using NanoString technology.

**Results:**

Peripheral clock activity was detected, regardless of prematurity and birth weight classification. The mean period, amplitude, and phase of circadian oscillations were not significantly associated with gestational age or birth weight classification.

**Conclusions:**

Peripheral clock activity can be demonstrated in HUVECs from both preterm and term infants without significant developmental differences in the period, amplitude, and phase of oscillations. This model may be useful to identify perturbation factors of proper development and entrainment of neonatal circadian clock activity.

**Impact:**

- We established a model system for analyzing the peripheral clock in preterm and term HUVECs.
- In HUVECs, the peripheral clock exhibits functional in vitro activity independent of gestational age or birth weight categories.
- In this model system, neither significant developmental differences exist in the period, amplitude, and phase, nor in the expression of circadian core clock and clock-associated genes.
- Entrainment and proper function of the circadian clock deserve attention in neonatal intensive care.

## Introduction

The function and impact of the circadian clock in human health and disease have been extensively investigated in recent years, mainly in adults.^1^ The circadian clock system is essential for the efficiency of metabolic, physiological, and behavioral processes and provides the body with resources in a time-of-day-adapted manner. Disruption of the circadian clock, e.g., by shift work, artificial light, travel across time zones, or social jetlag, has been associated with a variety of diseases, e.g., sleep disorders, cancer, psychiatric and cardiovascular diseases, chronic systemic inflammation, and impaired immune response.^2^ Disruption of sleep and circadian rhythms is also considered a risk factor for neurodevelopmental disorders and poor outcomes after (neonatal) intensive care.^3–5^

In mammals, the circadian system is organized in a hierarchic fashion, with the suprachiasmatic nucleus (SCN) of the anterior hypothalamus serving as the central pacemaker clock which synchronizes clocks in peripheral tissues through neuronal, neuroendocrine, and behavioral pathways (reviewed in ref. ^6^). Nevertheless, peripheral clocks have also been shown to be autonomous self-sustained oscillators, hinting at the existence of cell-specific synchronizers.^7–9^ The onset of central and peripheral circadian clock activities is considered a gradual process during mammalian ontogeny, but the exact time point of emergence is still under debate since it varies depending on the species and the experimental approach for detection.^10,11^ Current data indicate that the activity of the central clock begins in the last trimester of gestation, for example in rats not earlier than developmental stage E19, and in primates not before the third trimester.^12,13^ Correlation between maternal and fetal rhythms suggests a transplacental entrainment of fetal rhythms by maternal time cues such as hormones, nutrients, body temperature, or physical activity.^14–16^ It is unknown whether and to which extent the activity of peripheral circadian clocks varies through human gestation and upon pregnancy-associated disorders or antenatal treatment. From a perspective of neurodevelopmental outcome, this could be highly relevant, in particular in preterm infants as they are not only separated from transplacentally mediated time cues but also exposed to non-physiological circadian factors such as artificial lighting, noise, and feeding regimens during neonatal intensive care.^17,18^

While the expression of molecular core clock regulators, such as *CLOCK* and *PER1* mRNA, has been detected in cultured term human neonatal fibroblasts and keratinocytes,^19^ to the best of our knowledge, peripheral circadian clock gene activity has never been demonstrated in human neonates. In this explorative study, we have studied the onset and activity of the molecular clock in preterm and term neonates at the time of birth by characterizing oscillations in human vein endothelial cells (HUVECs) obtained from their umbilical cords.

## Materials and methods

### Subjects and samples

Subjects for the NeoCIRCLE (Assessing the Neonatal Circadian Clock and Entrainment Factors) study were recruited at the Department of Obstetrics after obtaining written informed consent (institutional review board approval no. EA2/171/22). Patients’ demographic data are summarized in Table 1 and Supplementary Data 2, respectively. Infants with congenital malformations, syndrome, or perinatal asphyxia were excluded.

**Table 1:** Demographic data and clinical characteristics of the study population, assigned to subgroups according to the subject’s birth weight. (*without ELBW infants).

Demographic data	Group 1 Birth weight >2500 g (*n* = 26)	Group 2 LBW 1500–2500 g (*n* = 18)	Group 3* VLBW 1001–1499 g (*n* = 7)	Group 4 ELBW ≤1000 g (*n* = 9)
Birth weight (g), median (range)	3342 (2515–4210)	1950 (1620–2460)	1170 (1100–1490)	700 (430–920)
Gestational age at birth (weeks + days), median (range)	38 + 4 (35 + 2–40 + 0)	33 + 5 (29 + 3–38 + 0)	29 + 1 (28 + 0–32 + 6)	24 + 6 (23 + 1–26 + 2)
Sex, female, *n* (%)	12 (46)	6 (33.3)	5 (71.4)	5 (55.6)
Twin child, *n* (%)	3 (11.5)	7 (38.9)	6 (85.7)	4 (44.4)
Antenatal glucocorticoids, *n* (%) ACT < 48 h before birth, *n* (%)	0	11 (61.1) 4 (22.2)	7 (100) 1 (14.3)	9 (100) 2 (22.2)
Mode of delivery, *n* (%)				
• Primary cesarean section	20 (76.9)	5 (27.8)	0	0
• Secondary cesarean section	4 (15.4)	5 (27.8)	6 (85.7)	7 (77.8)
• Vaginal	2 (7.7)	8 (44.4)	1 (14.3)	2 (22.2)
Gestational and perinatal disorders, *n* (%)				
Intrauterine growth restriction	0	2 (11.1)	3 (42.9)	2 (22.2)
Premature rupture of membranes or placenta praevia bleeding	3 (11.5)	7 (38.9)	3 (42.9)	7 (77.8)
Maternal diabetes (gestational, type I, type II)	4 (15.3)	1 (5.6)	0	0
Early-onset infection (diagnosed by IL-6 plasma concentration at birth >100 pg/mL)	2 (7.6)	2 (11.1)	2 (28.6)	5 (55.6)

### Isolation and culture of primary HUVECs

Umbilical cord specimens were obtained immediately after birth and stored in Hank’s Balanced Salt Solution (HBSS, Thermo Fisher, Henningsdorf, Germany, #14175053) at 4–8 °C for a median of 2 h (95% confidence interval (CI) 1.1–2.9 h) until isolation. For isolation, the umbilical vein was rinsed with HBSS, instilled with 0.5 mg/mL collagenase A solution (Roche Diagnostics, Mannheim, Germany, #10103586001) in phosphate-buffered saline (PBS, Thermo Fisher, #14190-094), gently massaged and incubated at 37 °C for 15 min immersed in water. After another gentle massage, the HUVECs were rinsed from the umbilical vein drop-by-drop with 40 mL HBSS and collected in a tube filled with 10 mL Medium 199 (Thermo Fisher, #31153026). Isolated cells were pelleted at 788 × *g* for 10 min, resuspended and cultured in Medium 199 supplemented with 20% heat-inactivated fetal bovine serum (FBS, Sigma–Aldrich, St. Louis, MO, #F7524), 2 mM L-glutamine (Bio&Sell, Feucht, Germany, #BS.K0283) and 20.7 mM NaHCO_3_ (Bio&Sell, #BS.L1713). In the following, this composition is referred to as “Medium 199 s”. The next day, Medium 199 s was exchanged to remove erythrocyte-containing residues. To attain proliferation, 19.95 µg/ml endothelial growth factor (ECGF, Biozol, Hamburg, Germany, #CYT-026948) and 15 U/mL heparin (Ratiopharm, Ulm, Germany, #X01336A) were directly added to the culture dish. Cultures were kept at 37 °C and 5% CO_2_ and expanded up to four passages (mean:15.5 d, range: 11–22 d) until bioluminescence recording.

### Immunocytological staining of HUVECs

The endothelial origin of the cells was confirmed by immunocytological staining of the exemplary cell pools for the endothelial marker protein platelet endothelial cell adhesion molecule 1 (PECAM1/CD31). HUVEC isolates were grown on fibronectin-coated (Sigma–Aldrich, #F2006-1MG; 0.031 mg/mL) chambered coverglass slides (Thermo Fisher, #177445) and fixed with 10% Roti Histofix (Roth, Karlsruhe, Germany, #A146.6) at a density of 7.5 × 10^4^ cells/cm^2^. Cells were permeabilized with 0.5% Triton X-100 (Sigma–Aldrich, #T9284) for 30 min and blocked with 5% skimmed milk in PBS for 1 h. The target protein was detected with a polyclonal rabbit anti-PECAM1 antibody (Abcam, Cambridge, UK, #ab28364), diluted 1:50 in antibody diluent (Invitrogen, Thermo Fisher, #00-3118) and incubated at 4 °C. The next day, a secondary Alexa Fluor® 488-coupled goat anti-rabbit IgG antibody (Jackson ImmunoResearch, PA, #111-545-003; 1:200) was added for 1 h at room temperature. Polyclonal rabbit IgG (PeproTech, Hamburg, Germany, #500-00; 1:50) served as a negative control, and cell nuclei were counterstained with Hoechst 33342 (Sigma–Aldrich, #14533) at a final concentration of 0.01 mg/mL. Results were observed under a fluorescent microscope (Keyence BZ-900, Neu-Isenburg, Germany). Images were taken with the built-in camera and processed with BZ-II-Viewer and BZ-II-Analyzer (Keyence). All samples tested (term: 38 + 4, LBW: 34 + 1, ELBW: 26 + 1 weeks gestation) showed >98% PECAM1-positive cells (Supplementary Data 1).

### Lentiviral transduction

**Fig. 1:**
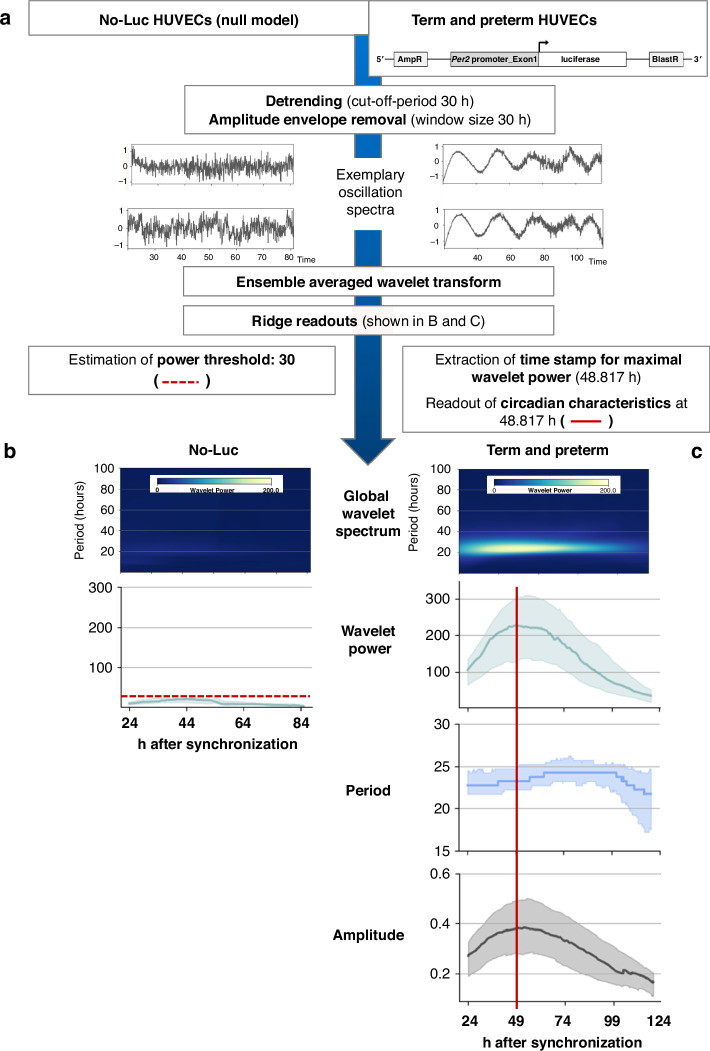
Extraction of oscillatory characteristics with the software pyBOAT. **a** Steps before the final analysis of recorded time-series data included pre-processing of all signals and ensemble-averaged rhythmical feature extraction of the entire data set of term and preterm samples vs. non-transduced (No-Luc) samples that served as null models (control). A schematic drawing of the lentiviral *Per2*prom-driven luciferase reporter can be found in the top right corner. AmpR: Ampicillin resistance gene, BlastR: Blasticidin resistance gene. **b** PyBOAT creates plots of the median and the interquartile range for each oscillatory characteristic over time (wavelet power, period, amplitude) for No-Luc and **c** for specimens from preterm and term infants. The point of maximal wavelet power (48.817 h post dexamethasone synchronization, red line) was used to extract oscillatory characteristics at a time point with the best data quality with regards to culture condition, duration of cell synchronization, and cell vitality as the ensemble-averaged data showed a decline of wavelet power, amplitude strength and a larger dispersion of period lengths with increased recording time.

Human embryonic kidney 293T cells (DSMZ, Braunschweig, Germany), cultivated in Dulbecco’s modified Eagle’s medium/Ham’s F12 (1:1, Corning, Corning, NY, #10-090-CV), supplemented with 10% FBS, were plated on T75 flasks at 5 × 10^4^ cells/cm^2^. Cells were transiently transfected with 7.5 µg DNA containing 3.375 µg of the lentiviral vector pLenti6.4_mPer2promExon1-Luc (a kind gift of Andrew Liu, first described in ref.^20^, Fig. 1), 2.625 µg psPAX2 (Addgene #12260), 1.5 µg pMD2.G packaging vectors (Addgene #12259) and 22.5 µl Fugene® 6 Transfection Reagent (Promega, Fitchburg, WI, #E2691) in Medium 199 s. The virus-containing supernatant was collected after 24 and 48 h of transfection, pooled, and either snap-frozen for storage at −80 °C or directly used for transduction of HUVECs. Then, HUVECs were seeded on 60 mm dishes at 5.25 × 10^4^ cells/cm^2^ (75% confluency) and incubated with 5 mL virus-containing supernatant for 24 h under standard cell culture conditions. After removal of the virus, the samples were selected for stably transduced cells using 6 µg/mL blasticidin S (Fisher Scientific, #10264913) at a cell density of <6.3 × 10^4^ cells/cm^2^ (approx. 90% confluency) for 3 days. Cells were then either stored in liquid nitrogen (*n* = 40) or directly underwent bioluminescence recording (*n* = 20). We did not observe differences in growth, transduction efficiency, or bioluminescence recording quality between freeze-thawed and fresh HUVEC samples, which were evenly distributed amongst the study groups.

### Cell synchronization and bioluminescence recordings

HUVECs were seeded into fibronectin-coated 35 mm dishes (Thermo Fisher, #150318) at a near-confluent density of 7 × 10^4^ cells/cm^2^. After a minimal period of 16 h and prior to bioluminescence recordings, cell rhythms were synchronized following an established protocol.^21^ In short, 1 µM dexamethasone (Sigma–Aldrich, #D4902-25MG, 1 mM stock in ethanol) was added to the media, and the sample was incubated for 20 min. After washing with PBS, Medium 199 without phenol red (Thermo Fisher, #11043023) was added, supplemented as Medium 199 s with addition of 19.95 µg/ml ECGF and 15 U/mL heparin, 1% penicillin-streptomycin (Bio&Sell, #BS. A 2212), 20 mM HEPES (Bio&Sell, #BS. L 1613) and 0.25 mM D-luciferin (PJK, Kleinbittersdorf, Germany, #102113). The samples were measured in a tailor-made luminometric system consisting of lightproof boxes kept at 36 °C and 5% CO_2_ in a standard cell culture incubator without humidity, connected to highly sensitive photomultiplier tubes (H7360-02, Hamamatsu Photonics, Hamamatsu, Japan). Live bioluminescence was recorded in at least technical duplicates for 5–7 days. Cell cultures were microscopically inspected for contamination and vitality at the end of each recording.

### RNA extraction and expression analysis of core clock and clock-associated genes

Different HUVEC samples were seeded at a density of 7 × 10^4^ cells/cm^2^ on 12-well plates (Corning, #353043) in 8 replicates each, one per time point. All samples were synchronized in parallel with 1 µM dexamethasone for 20 min and washed with PBS before the media was exchanged to fresh Medium 199 s. RNA was extracted from each well with 500 µL TRIzol Reagent (Thermo, #15596018) at 24, 28, 32, 36, 40, 44, 48, and 52 h after synchronization, according to the manufacturer’s instructions.

Expression quantification of 20 clock and clock-associated genes (*ARNTL2, BHLHE40, BHLHE41, BMAL1, CIART, CIPC, CLOCK, CRY1, CRY2, CSNK1D, CSNK1E, DBP, NFIL3, NPAS2, NR1D1, NR1D2, PER1, PER2, PER3, RORA*) was performed from HUVEC RNAs obtained from different gestational ages as well as different culture times after isolation by NanoString technology as described.^22^ To evaluate whether the average gene expression levels associated with birth weight classification, the HUVECs were synchronized by dexamethasone treatment (1 µM, 20 min) and sampled as described above. To obtain mean gene expression values, the resulting RNAs were averaged by pooling equal RNA amounts from each time point. For determining the influence of the culture time since isolation, RNA of HUVEC samples was pooled according to maturity and birth weight classification and culture time (6/8/10/15.5 d after preparation). Gene expression in the different groups was tested for normality (all log-normal) and the log10 values were analyzed for statistical differences by one-way ANOVA.

### Data analysis

For data analysis, all time-series data were cropped to the recording time frame of 24–120 h. The software pyBOAT was used - a wavelet-based time-frequency analysis software, specifically designed for non-stationary and noisy rhythmical data.^23^ In brief, the signal was pre-processed by detrending and amplitude envelope removal. The recorded signal was then decomposed into different frequency components across the recording period by applying the wavelet transformation, and its time-frequency representation was visualized as the wavelet power spectrum displaying the main oscillatory component of the signal. Ridge tracking allowed extraction of the rhythm parameters amplitude, period, and phase. The user-defined filter parameter “Cut-Off-Period” was set to 30 h, so that the program removed low-frequency signals commonly induced by non-circadian cellular processes like cell division. Signals were normalized with an amplitude envelope estimated from a window size of 30 h.

To distinguish oscillating from non-oscillating signals, we estimated a wavelet power threshold for the background noise of a null model established from bioluminescence recordings of non-transduced HUVECs (in the following referred to as No-Luc). For this, 14 No-Luc samples underwent the same procedures as described above apart from the viral transduction and selection step and were recorded for 120 h. An ensemble-averaged wavelet analysis for (a) all No-Luc and (b) all term and preterm (samples of interest) recordings was performed, which showed that 48.817 h post dexamethasone synchronization was the point of maximal wavelet power (median of the batch analysis). This timestamp was used to extract oscillatory characteristics at a time point with the best data quality regarding culture condition, duration of cell synchronization, and cell vitality. Samples with a wavelet power <30 (mean + 2 SD of the No-Luc samples) were categorized as non-oscillating (Fig. 1). A mean absolute bioluminescence <6700 (mean + 2 standard deviations of the No-Luc samples) was used as a minimal brightness threshold, and recordings below were excluded from the analysis.

Oscillatory parameters at 48.817 h were first tested for outliers by Grubbs’ test (one value for phase removed from the >2500 g/≥37 wks and 2500–1500 g/36 + 6–32 + 0 wks cohorts, respectively), then tested for normality by Shapiro–Wilk test (all normal or log-normal) and analyzed for statistical differences between the groups with a BW > 2500 g, 2500–1500 g, 1499–1000 g and <1000 g, or between groups of term (≥37 + 0 wks gestation), preterm (32 + 0–36 + 6 wks gestation), very preterm (28 + 0–31 + 6 wks gestation) and extremely preterm infants (<28 + 0 wks gestation), respectively, by one-way ANOVA and correlation analysis with a significance threshold of *p* < 0.05 (GraphPad Prism 9, GraphPad Software Inc., San Diego).

## Results

### Study population

**Fig. 2:**
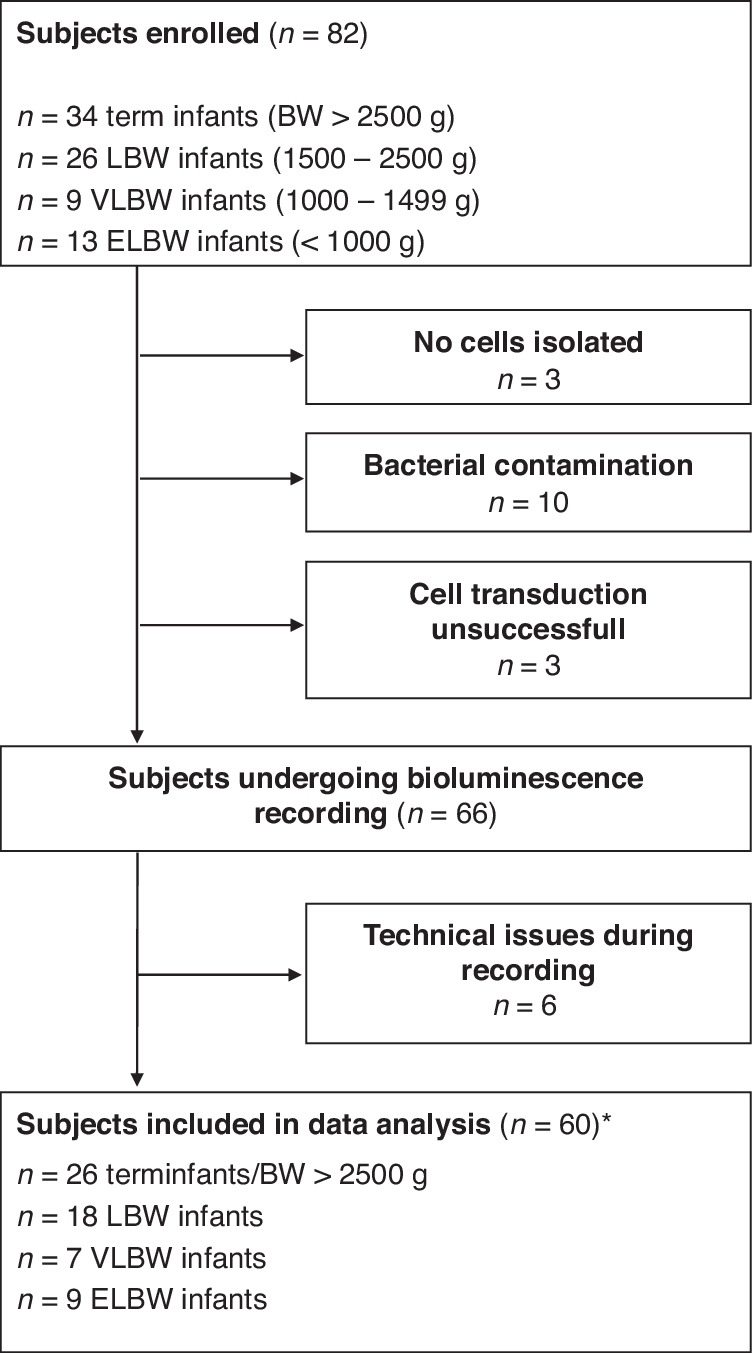
Flow chart of the NeoCIRCLE study. BW birth weight, LBW low birth weight, VLBW very low birth weight, ELBW extremely low birth weight. * For 7 samples, only one replicate of the recording data could be included.

From 2022 to 2023, 82 term and preterm neonates were enrolled in the NeoCIRCLE study, equal to 82 HUVEC samples, 22 of which had to be excluded from the final analysis due to: Unsuccessful cell isolation (*n* = 3), bacterial contamination (*n* = 10), inefficient lentiviral transduction (*n* = 3) or technical issues during recording (*n* = 6), resulting in a total of 60 subjects. The final study cohort was subgrouped according to BW (Fig. 2). Clinical conditions were reflected within the BW-specific groups as expected: Gestational or perinatal disorders and secondary cesarean sections were more common in the subgroups of preterm infants that also exhibited a higher proportion of twin births and frequency of antenatal glucocorticoid (GC) treatment (Table 1). The underlying clinical conditions showed almost equal distributions if the final study cohort was subgrouped according to GA (Supplementary Data 2).

### A peripheral clock oscillates in term and preterm HUVECs at the time of birth

**Fig. 3:**
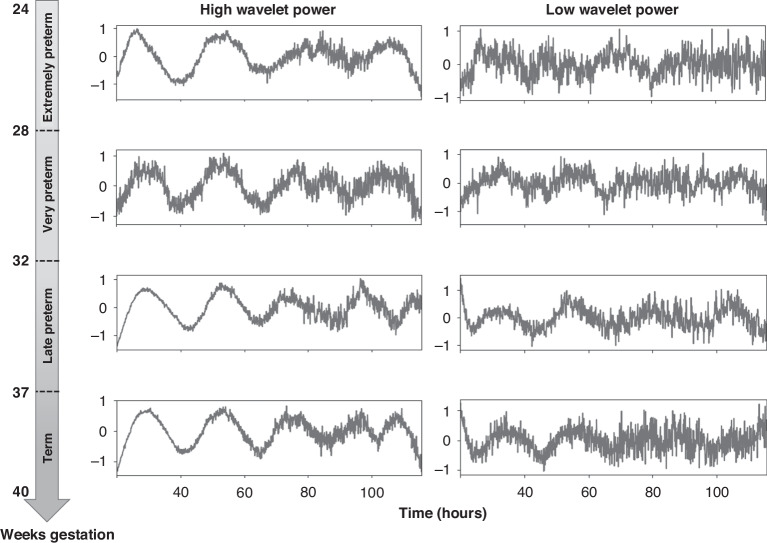
Oscillation curves of HUVEC samples of different gestational ages. Representative bioluminescence oscillation curves with high and low wavelet powers are shown for subjects of different gestational ages and birth weights, detrended and amplitude-normalized with the pyBOAT software.

To determine whether the functional activity of the clock exists during fetal development and if so, might depend on birth weight (BW) or gestational age (GA), respectively, we obtained HUVECs at birth of infants born at 23 + 1 to 40 + 0 weeks gestation (birth weight 430–4210 g). Cells were in vitro transduced with a luciferase reporter gene construct under circadian control. After restarting the intercellular clocks with a synchronizing dexamethasone pulse, the resulting bioluminescence was live-recorded over a period of five days. When processed by the analysis software pyBOAT, recordings showed oscillations of weak and moderate signal strength at all stages of development (Fig. 3, all 60 recording tracks can be found in Supplementary Data 3).

**Fig. 4:**
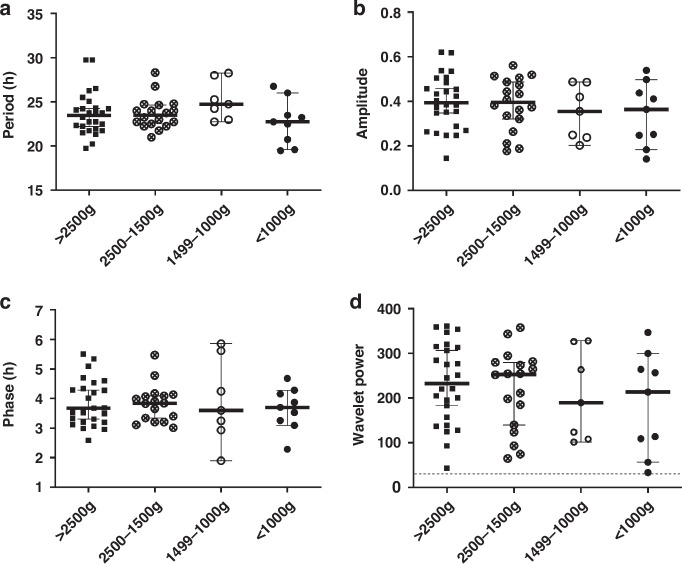
Oscillatory characteristics of HUVECs from preterm and term neonates by birth weight. Plots show the median and 95% CI of infants with a birth weight >2500 g (black square, *n* = 26) vs. 2500–1500 g (crossed circle, *n* = 18) vs. 1499–1000 g (open circle, *n* = 7) vs. <1000 g (black circle, *n* = 9) for the oscillatory characteristics period (**a**), amplitude (**b**), phase (**c**), and the oscillatory quality control parameter wavelet power (**d**) at the time point of maximal wavelet power (48.817 h after dexamethasone synchronization). The dashed line in (**d**) represents the minimal wavelet power threshold of 30 to be considered a “true” oscillation. Wavelet power values of all samples were above this threshold classifying them as “real” oscillations.

**Fig. 5:**
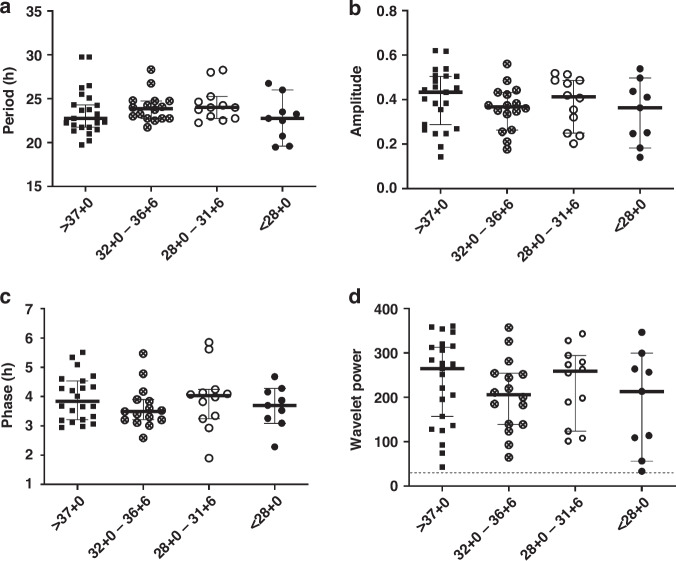
Oscillatory characteristics of HUVECs from preterm and term neonates by gestational age. Plots show the median and 95% CI of infants with gestational age at birth ≥37 wks (black square, *n* = 26) vs. 32 + 0–36 + 6 wks (crossed circle, *n* = 18) vs. 28 + 0–31 + 6 wks (open circle, *n* = 7) vs. <28 wks (black circle, *n* = 9) for the oscillatory characteristics period (**a**), amplitude (**b**), phase (**c**), and the quality control parameter wavelet power (**d**) at the time point of maximal wavelet power (48.817 h after dexamethasone synchronization). The dashed line in (**d**) represents the minimal wavelet power threshold of 30 to be considered a “true” oscillation. Wavelet power values of all samples were above this threshold classifying them as “real” oscillations.

When comparing the oscillation quality of preterm and term specimens by the oscillatory quality control parameter wavelet power, those were similar in all four BW groups (>2500 g: 233 ± 90, 2500–1500 g: 219 ± 89, 1499–1000 g: 206 ± 100, <1000 g: 188 ± 113, one-way ANOVA: *p* = 0.647), all exceeding the threshold for background noise of 30 (Fig. 4, dotted line). A mean period of about 23–25 h in all subgroups (>2500 g: 23.7 ± 2.5 h, 2500–1500 g: 23.7 ± 1.8 h, 1499–1000 g: 25.2 ± 2.2 h, <1000 g: 22.7 ± 2.6 h, one-way ANOVA: *p* = 0.183) indicated that the oscillations were indeed of circadian nature, and did not largely differ between the BW-specific groups, even if the period in the ELBW group tended to be lower than that of the other groups (Fig. 4a). The amplitude as a surrogate of the oscillation strength was similar in all subgroups (>2500 g: 0.40 ± 0.12, 2500–1500 g: 0.39 ± 0.12, 1499–1000 g: 0.35 ± 0.12, <1000 g: 0.34 ± 0.14, one-way ANOVA: *p* = 0.591, Fig. 4b), but revealed a trend in HUVECs from VLBW infants towards lower amplitude oscillations than in (near-)term infants (>2500 g: 0.40 ± 0.12 vs. <1500 g: 0.34 ± 0.13). Finally, the parameter phase indicates the relative position within one oscillatory cycle and represents the sample’s reaction to the synchronization. This phase was similar in all subgroups (>2500 g: 3.9 ± 0.8 h, 2500–1500 g: 3.8 ± 0.6 h, 1499–1000 g: 3.9 ± 1.4 h, <1000 g: 3.8 ± 0.7 h, one-way ANOVA: *p* = 0.915, Fig. 4c), showing neither delay nor advance between the oscillation tracks of term and preterm samples. Of note, the results were similar if the samples were subgrouped by GA instead of BW (Fig. 5). A correlation analysis of the circadian parameters within the BW and GA groups, respectively, revealed a (technically expected) significant correlation between amplitude and wavelet power in all groups. Of note, there also was a significant correlation between period and amplitude as well as period and wavelet power, respectively, in the group with a BW < 1000 g. All correlation results can be found in Supplementary Data 4.

**Fig. 6:**
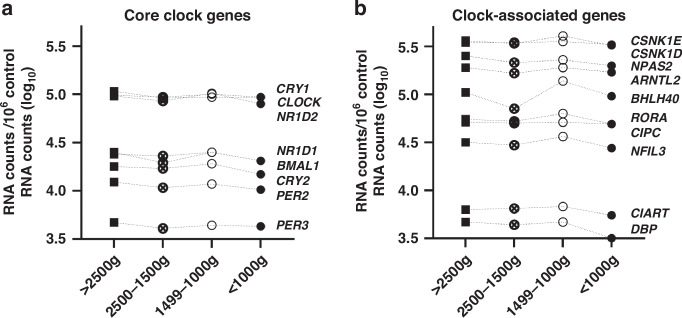
Mean expression of core clock and clock-associated genes in HUVECs from preterm and term neonates by birth weight. Mean expression of 8 core clock (**a**) and 10 clock-associated genes (**b**) normalized to four control genes (*GAPDH*, *PPIA*, *HPRT1*, *PSMB2*) in HUVEC samples from infants with a BW > 2500 g (*n* = 6), 2500–1500 g (*n* = 4), 1499–1000 g (*n* = 3) and <1000 g (*n* = 4). Cells were synchronized and sampled over a 24–52 h time period, then averaged by pooling equal RNA amounts from each time point. The mean gene expression was not statistically different between subgroups classified according to birth weight. Dotted line combine data points of the same gene product. The complete data set can be found in Supplementary Data 5.

To test whether there were subtle differences in the expression of core clock and clock-associated genes, we analyzed the expression of 18 out of a panel of 20 genes (*BHLHE41* and *PER1* expression were below the detection threshold) in synchronized HUVEC cultures of different BW groups pooled from a 28 h time course each (Fig. 6). From a statistical point of view, there were no significant differences, but some genes displayed a trend towards lower expression in preterm samples (Fig. 6, shaded in gray in Supplementary Data 5): the core clock genes *PER2*, *CRY1*, *NR1D1*, *NR1D2*, and the clock-associated genes *ARNTL2 (BMAL1* paralogue) and *NPAS2* (*CLOCK* paralogue).

Taken together, we found that HUVECs had the capacity of basal circadian oscillations at the time of birth, irrespective of whether the infants were born prematurely or at term, but with a non-significant trend towards lower amplitudes and periods in VLBW and especially ELBW infants.

## Discussion

In this study, we show for the first time circadian oscillations of the peripheral clock in primary HUVECs isolated from the umbilical cord of preterm and term infants (Figs. 3–5). Thus, in future studies, our model system may allow a detailed description of factors that could modify the activity of the endogenous peripheral clock during development (e.g. ischemia, metabolic or inflammatory factors).

We were also able to portray clock properties in HUVECs of term vs. preterm infants, and show that endogenous, cell-autonomous (PER2-mediated) clock activity already existed in vitro and that oscillation quality did not differ significantly between the BW- or GA-classified subgroups (Figs. 4 and 5). Neither maturity (and BW classification) nor culture time since isolation significantly affects the expression of the core clock and clock-associated genes (Fig. 6, Supplementary Data 5 and 6). However, period and amplitude and the average expression of some of the core clock and clock-associated genes tended to be lower in the groups with a BW < 1500 g. This might indicate that there are indeed subtle differences in the circadian parameters, but that statistical discriminability is limited by the relatively small sample number. Consistent with this observed trend in the circadian profile of HUVECs, a very recent publication showed circadian rhythm development of the neonatal heart rate in 66 preterm infants (29.8 ± 3.7 wks gestation) over the course of postnatal development.^24^

Experimental data using peripheral tissues of fetal mice (E18) showed rhythmicity in clock gene expression in culture, but not in vivo.^25^ Thus, one limitation of our model system could be that the GC dexamethasone was used for cell synchronization prior to bioluminescence recordings. Peripheral tissues and cells lose clock synchrony over time due to a lack of intercellular coupling which results in low signal-to-noise ratios and dampened rhythms.^9^ Therefore, we have chosen the most commonly used synchronization method of a dexamethasone pulse. The alternative synchronization method, cold shock, did not synchronize the HUVECs as well. Serum shock was excluded as we did not want to present the HUVECs with additional fetal plasma components with specific cytokine and metabolite compositions as potential perturbation factors for the developing clock.^26^ In addition to this in vitro synchronization pulse of dexamethasone, antenatal exposition of premature infants to synthetical GCs is common in the clinical routine - all ELBW, VLBW, and 70% of the LBW infants of our study population received betamethasone for antenatal GC treatment to improve neonatal outcome (Table 1).^27^ Maternal GCs are attributed to a synchronizing function in the growing fetus, and it is hypothesized that they are a key signal for the development and maturation of the fetal circadian system *in utero*.^28,29^ Thus, we cannot exclude that the observed (PER2-mediated) HUVEC rhythmicity was (partially) induced by the common antenatal GC treatment due to impending prematurity, but this would have only influenced the relative comparison with the most mature group as all others were similarly exposed to antenatal GCs.

Our analysis of circadian clock activity revealed that the mean period, amplitude, and phase of circadian oscillations in HUVECs were not significantly associated with BW or GA classification (Figs. 4 and 5), even if period and amplitude and some core clock and clock-associated genes tended to be lower in more preterm groups (Figs. 4–6). Regulation of core clock and clock-associated genes could be mediated by epigenetic modifications. It has been shown that in placental tissue and umbilical cord leukocytes, disorders such as early-onset preeclampsia rather than gestational age are associated with such CpG modifications.^30^ These combined findings suggest that the developing circadian system of premature infants will react differently to external (e.g., oxidative, metabolic, inflammatory) stressors present in neonatal intensive care. This is a novel and important finding, not adequately considered in current clinical practice. Yet, only very few studies have investigated potential rhythm patterns in very and extremely preterm neonates (26–32 weeks gestation), mostly focusing on vital parameters like body temperature, heart rate, and activity or motility during the early neonatal period. No strong evidence for circadian patterns in these parameters was found, and ultradian rhythms, probably influenced by interventions and feeding, appeared to mask putative circadian rhythms.^31–33^ Bauer et al. found diurnal variability in oxygen consumption (VO_2_, defined as a 5% fluctuation over baseline values) in 18 of 22 preterm subjects born at 27–31 weeks’ gestation, studied at 3–4 weeks’ postnatal age, suggesting the presence of central endogenous circadian activity in vivo.^34^ Although our HUVEC in vitro model may not fully reflect the developing internal clock in vivo, we were able to characterize the activity of the molecular clock independent from external factors that may induce ultradian rhythms or disrupt rhythmicity through clinical conditions like mechanical ventilation, sedation, or inflammation.

During intensive care, the circadian system of adults is often dysregulated, as e.g. clock gene expression was found down-regulated in septic and critically ill patients,^22,35^, and melatonin secretion was desynchronized in mechanically ventilated patients.^36^ At the same time, the circadian system seems to influence the course and outcome of critical conditions, such as ventilator-induced lung injury^37^ or the development of delirium.^38^ During the last decade, the concept of promoting circadian rhythms, in the meaning of “chronofitness”,^4^ to improve weight gain, comfort, and neurodevelopment of preterm and term neonates in intensive care has gained increasing attention.^3,5^ By detecting functional in vitro activity of the peripheral clock in HUVECs independent from gestational age, our data support further efforts to protect and entrain circadian rhythms even in the smallest and most premature infants. Which of the perturbation factors in the NICU might have the highest influence on the developing circadian system of preterm and term infants should be addressed in further in vitro studies using our HUVEC model system.

## Conclusions

We have established human umbilical vein endothelial cells as a model system for detecting peripheral clock activity by bioluminescence recording in primary cells of preterm and term neonates. In contrast to previous indirect clues from other species, there were no significant developmental differences in the period, amplitude, or phase of the peripheral clock in human endothelial cells which indicates that circadian activity exists independently from maternal signals and deserves attention during intensive care of very preterm neonates.

## Supplementary information

Supplementary Material

## Data Availability

The data used to support the findings of this study are available from the corresponding author upon request.
