# Supplementary material for: Circadian clock activity in human umbilical vein endothelial cells of preterm and term neonates
Source: Pediatr Res. 2024 Dec 13;98(2):734–42. doi: 10.1038/s41390-024-03705-3 (PMC12454134; doi:10.1038/s41390-024-03705-3)
Supplement: Supplementary file 1 — Supplementary Material [file 41390_2024_3705_MOESM1_ESM.pdf]

Supplementary Material

Supplementary Data 1: HUVEC PECAM1 immunocytochemistry

Supplementary Data 2: Clinical characteristics of the study cohorts by gestational age classification

Supplementary Data 3: All HUVEC recording tracks

Supplementary Data 4: Correlation results of circadian parameters within the birth weight and gestational age groups

Supplementary Data 5: Mean core clock and clock-associated gene expression values dependent on the HUVEC birth weight group

Supplementary Data 6: Core clock and clock-associated gene expression profiles over different culture times since HUVEC isolation

Supplementary Data 1

Term HUVECs

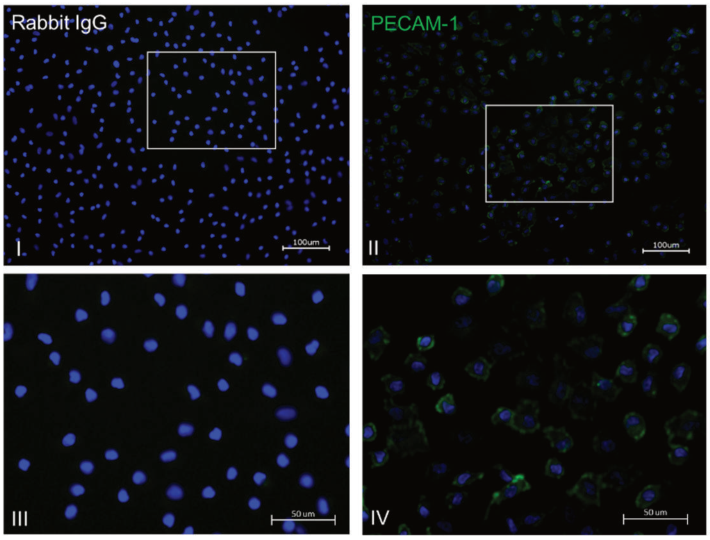

Late preterm HUVECs

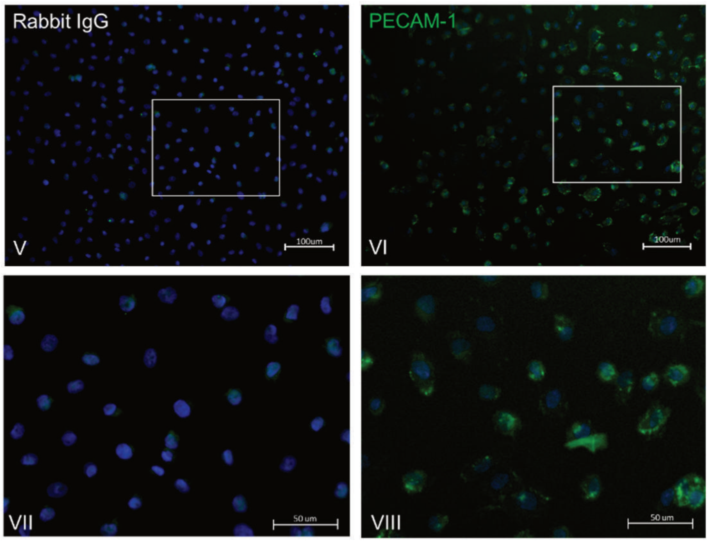

Extremely preterm HUVECs

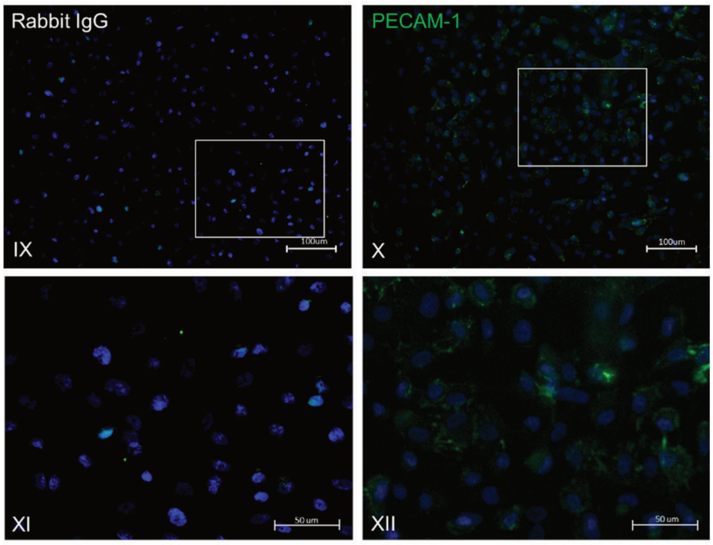

Supplementary Data 1: Exemplary immunocytological staining of cultivated primary HUVECs with endothelial marker protein Platelet and Endothelial Cell Adhesion Molecule 1 (PECAM1 / CD31). PECAM1-stained HUVECs of a representative term (38+4 wks gestation, **II/IV**), late preterm (34+1 wks gestation, **VI/VIII**), extremely preterm (26+1 wks gestation, **X/XII**) subject and the corresponding rabbit IgG negative control (**I/III/V/VII/IX/XI**). Cell nuclei were counterstained with Hoechst 33342, scale bars as indicated. All samples showed >98 % endothelial (PECAM1-positive) cells.

Supplementary Data 2: Demographic data and clinical characteristics of the study population, assigned to subgroups according to the subject's gestational age.

**Demographic Data**	**Group 1**<br>Term<br>≥ 37+0 wks<br>(n = 23)	**Group 2**<br>Late preterm<br>36+6 – 32+0 wks<br>(n = 16)	**Group 3**<br>Very preterm<br>31+6 – 28+0 wks<br>(n = 12)	**Group 4**<br>Extremely preterm<br>< 28+0 wks<br>(n = 9)
Birth weight (g), median (range)	3,380<br>(2,420 to 4,210)	2030<br>(1,100 to 3,225)	1,170<br>(1,100 to 1,490)	700<br>(430 to 920)
Gestational age at birth (weeks + days),<br>median (range)	38+4<br>(37+2 to 40+0)	33+6<br>(32+3 to 36+4)	29+5<br>(28+0 to 31+6)	24+6<br>(23+1 to 26+2)
Sex, female, n (%)	10 (43.5)	7 (43.7)	5 (41.7)	5 (55.6)
Twin child, n (%)	2 (8.7)	6 (37.5)	7 (58.3)	4 (44.4)
Antenatal glucocorticoid therapy (ACT), n (%)<br>ACT < 48 hours before birth, n (%)	0	6 (37.5)<br>1 (6.2)	12 (100)<br>4 (33.3)	9 (100)<br>2 (22.2)
Mode of delivery, n (%)<br>• Primary caesarean section<br>• Secondary caesarean section<br>• Vaginal	 21 (91.3)<br>0<br>2 (8.7)	 3 (18.7)<br>8 (50.0)<br>5 (31.2)	 1 (8.3)<br>7 (58.3)<br>4 (33.3)	 0<br>7 (77.8)<br>2 (22.2)
Gestational and perinatal disorders, n (%)<br>• Intrauterine growth restriction<br>• Premature rupture of membranes or placenta praevia bleeding<br>• Maternal diabetes (gestational, type I, type II)<br>• Early-onset infection (diagnosed by IL-6 plasma concentration at birth >100 pg/mL)	 0<br>0<br>2 (8.7)<br>2 (8.7)	 3 (18.7)<br>4 (25.0)<br>2 (12.5)<br>1 (6.2)	 3 (25.0)<br>7 (58.3)<br>0<br>3 (25.0)	 2 (22.2)<br>7 (77.8)<br>0<br>5 (55.6)

Supplementary Data 3

**No-Luc recordings**	
N066_Ch1 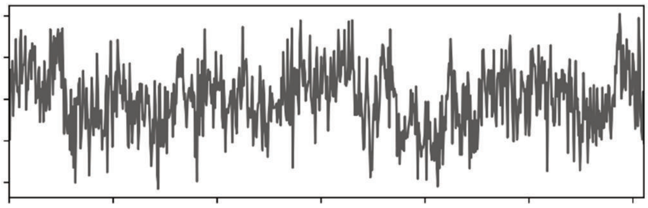	N072_Ch1 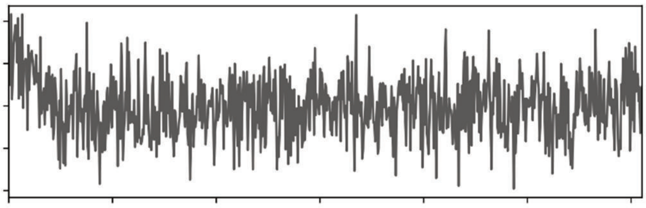
N072_Ch2 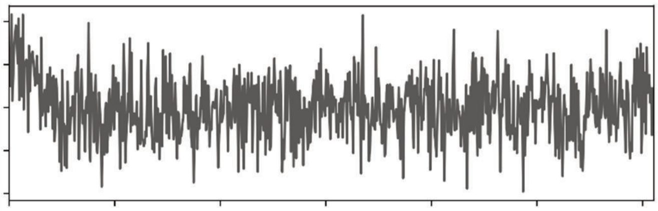	N075_Ch1 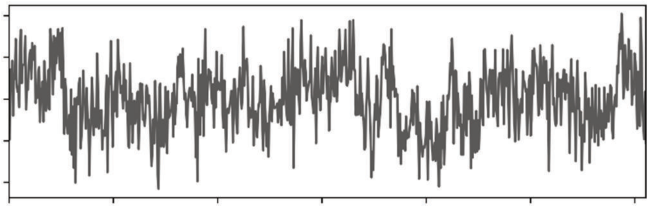
N075_Ch3 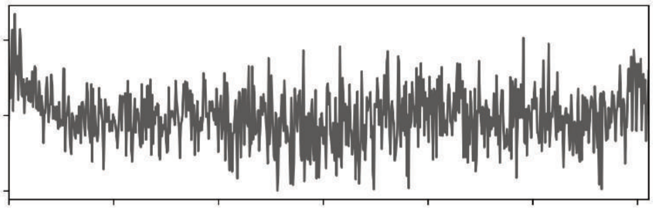	N075_Ch4 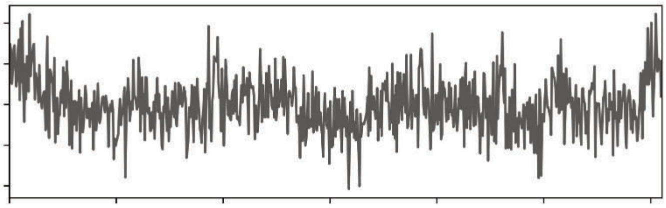
N075_Ch5 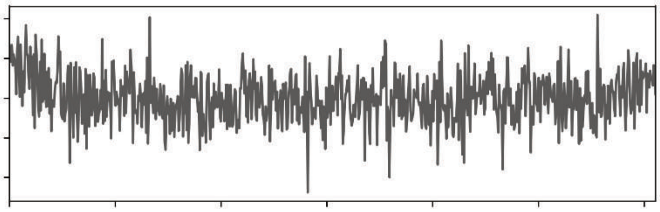	N075_Ch6 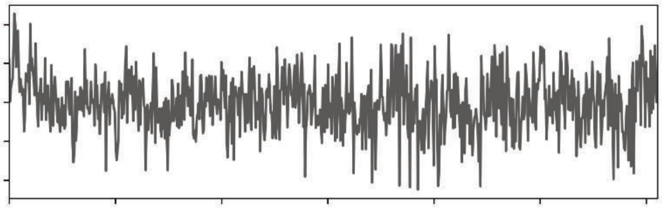
N075_Ch7 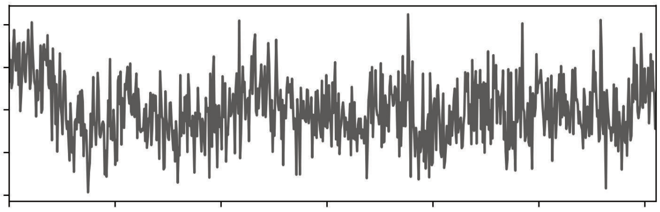	N075_Ch8 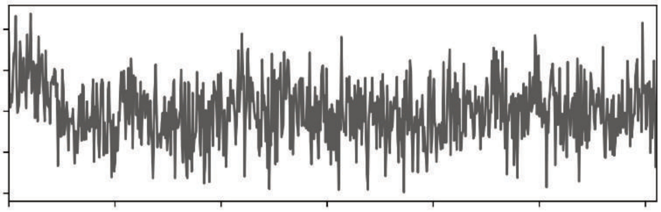
N089_Ch3 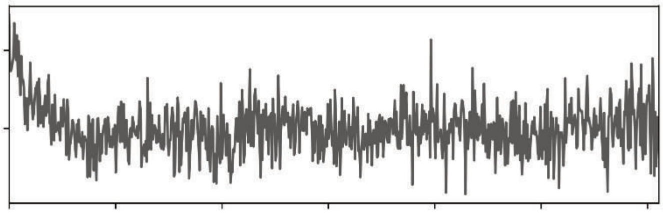	N089_Ch4 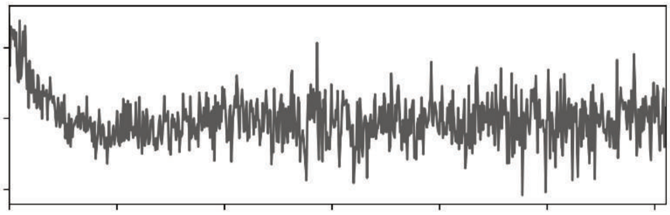

N089_Ch5<br>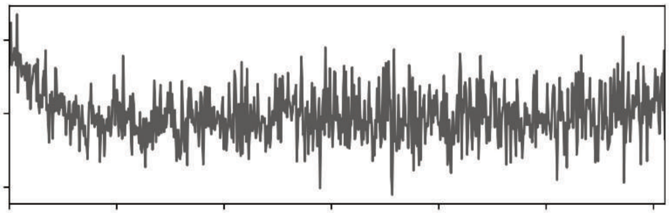	N089_Ch6<br>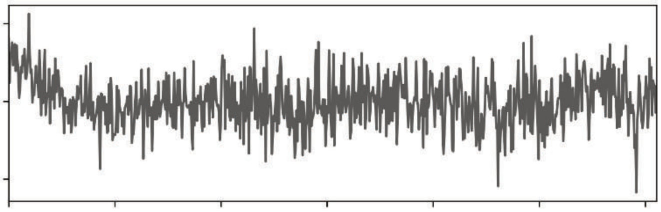
**Term recordings (n=26)**	
**Replicate 1**	**Replicate 2**
N001_Ch1<br>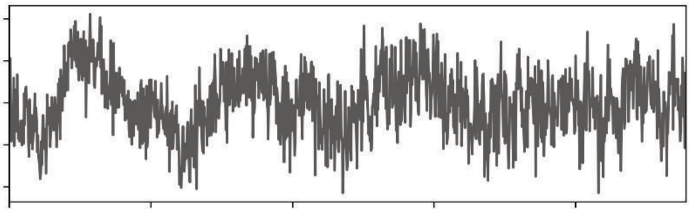	
N002_Ch3<br>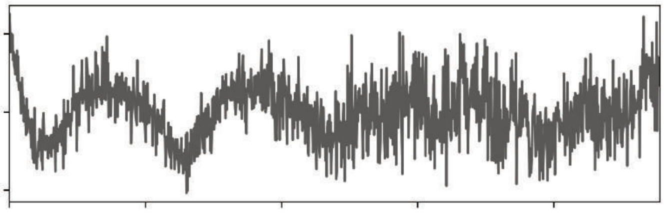	N002_Ch4<br>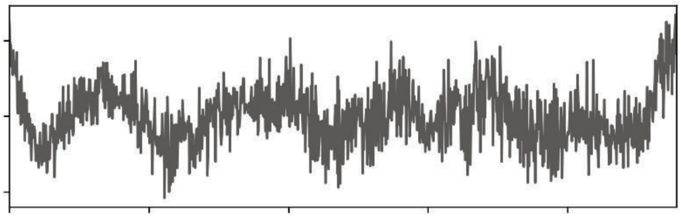
N016_Ch3<br>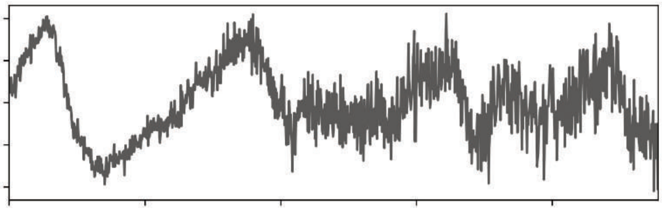	
N020_Ch3<br>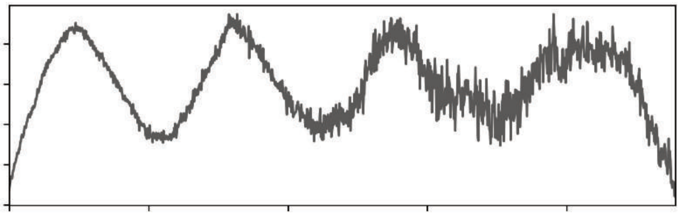	N020_Ch4<br>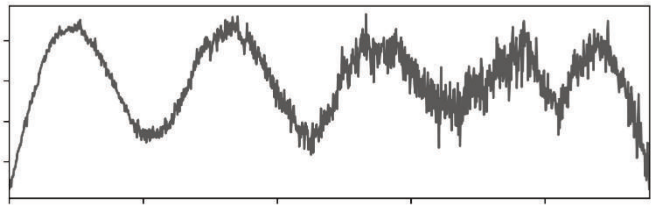
N029_Ch1<br>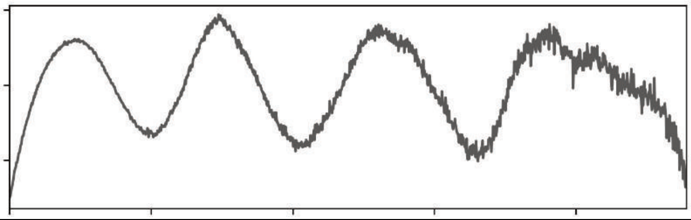	

N030_Ch2

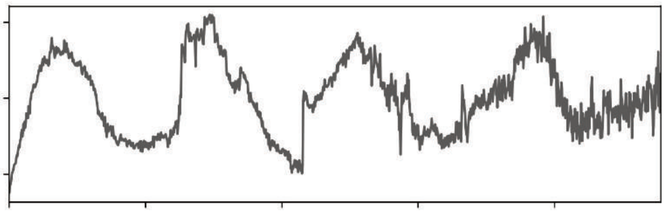

N030_Ch3

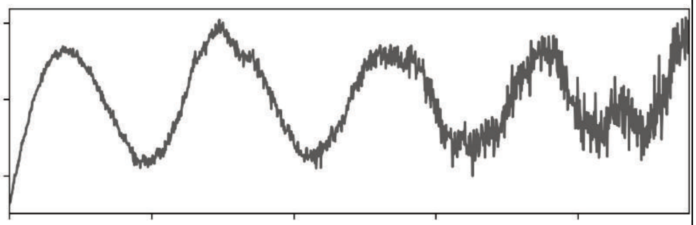

N031_Ch4

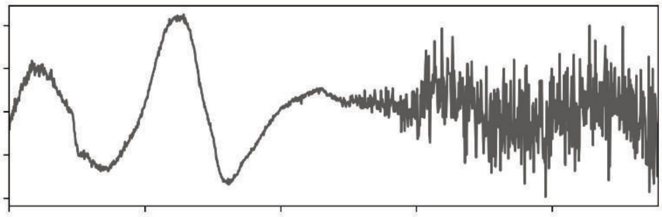

N032_Ch6

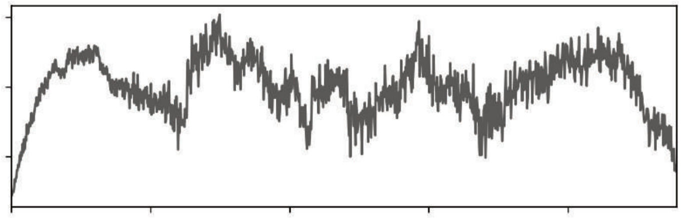

N032_Ch7

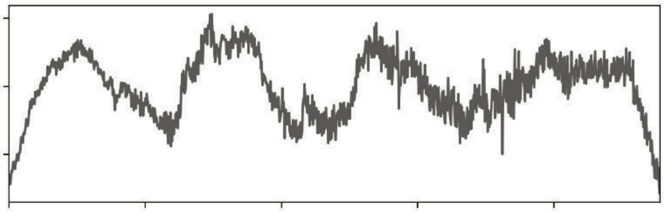

N039_Ch3

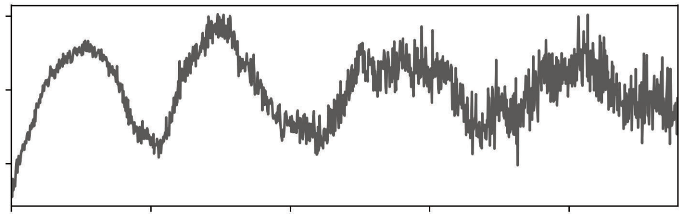

N039_Ch4

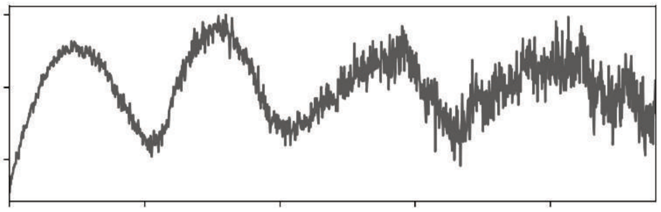

N040_Ch1

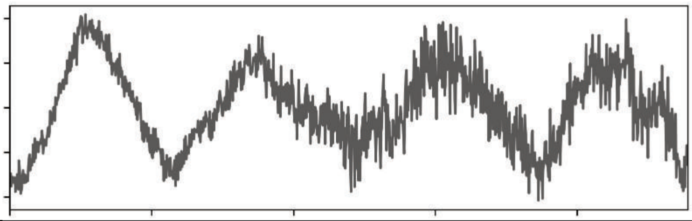

N040_Ch2

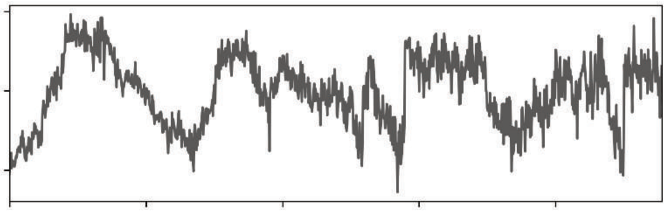

N041_Ch3

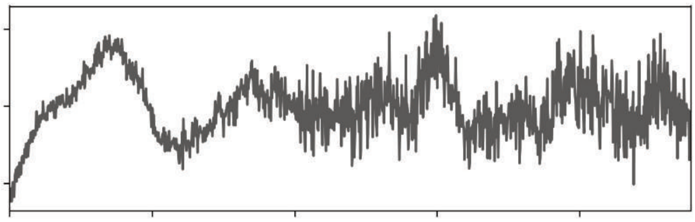

N041_Ch4

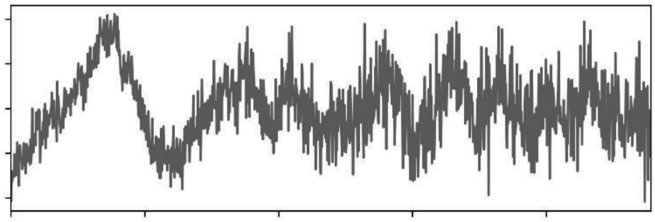

N043_Ch3

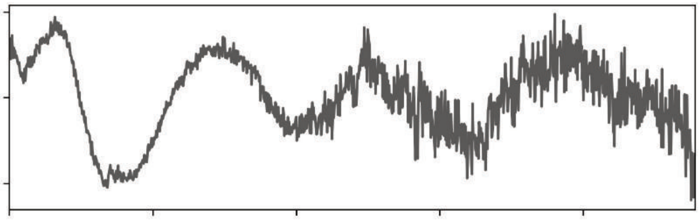

N044_Ch2 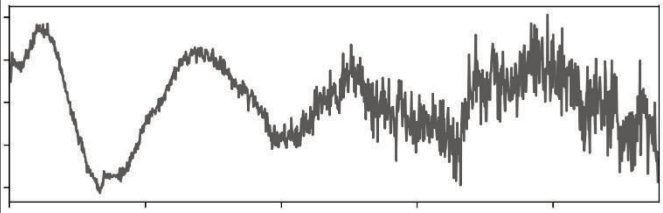	
N045_Ch1 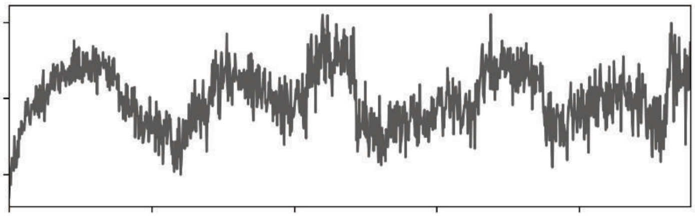	
N046_Ch2 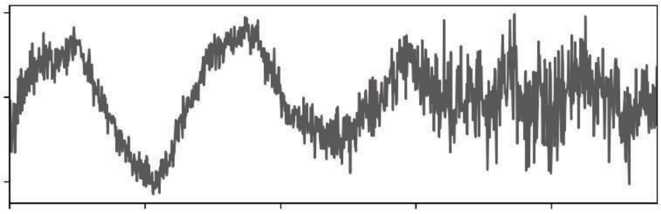	N046_Ch4 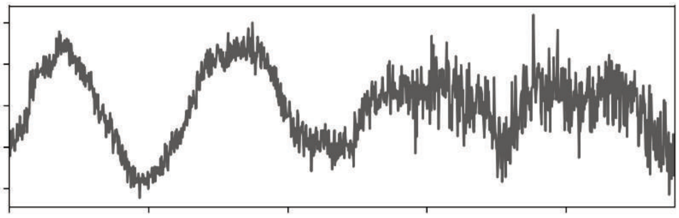
N058_Ch5 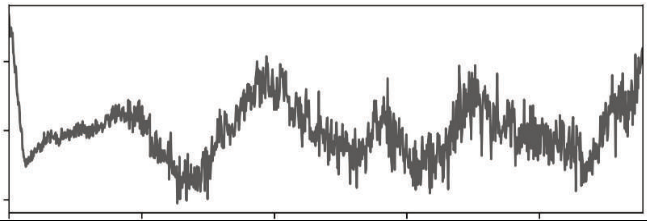	N058_Ch6 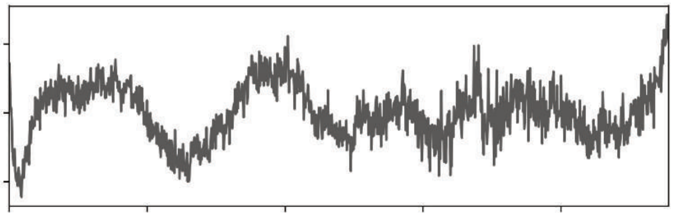
N059_Ch1 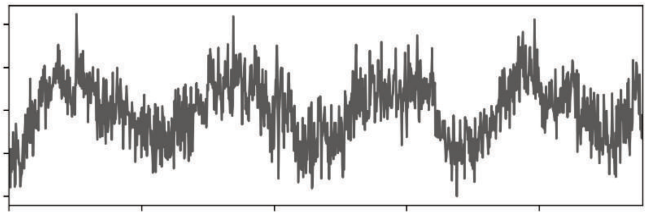	N059_Ch4 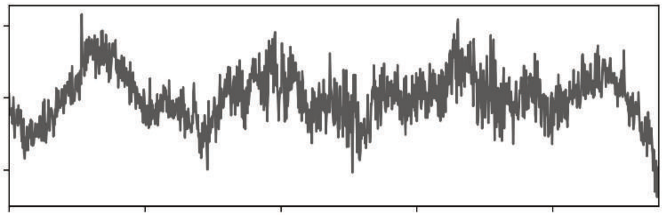
N062_Ch4 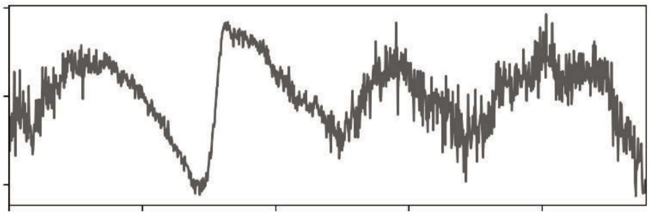	
N072_Ch1 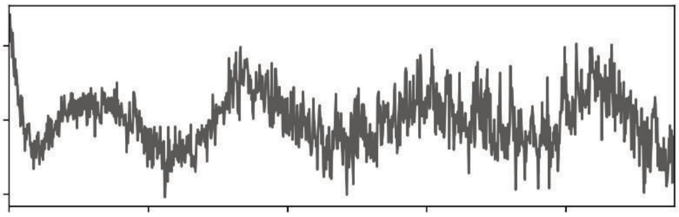	

N073_Ch6 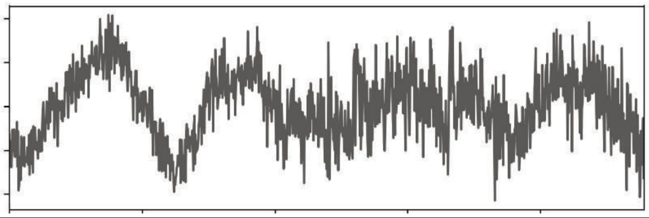	
N075_Ch6 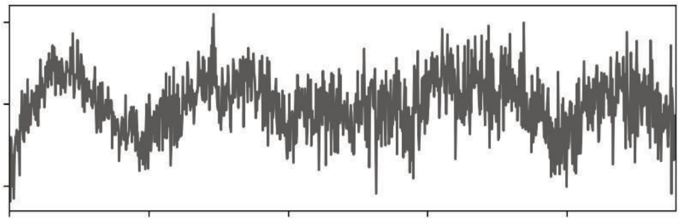	N075_Ch5 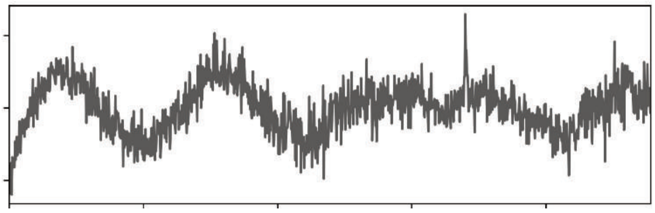
N077_Ch1 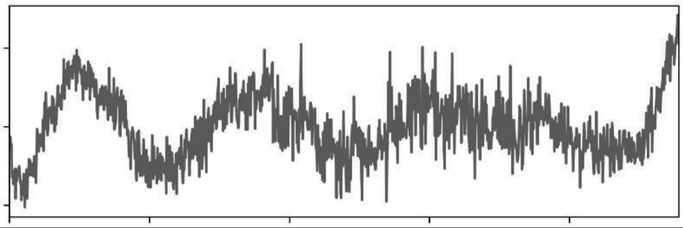	
N078_Ch4 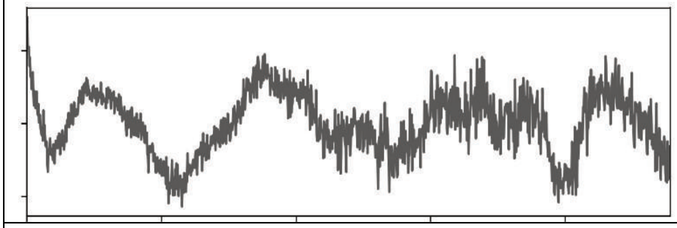	
N087_Ch4 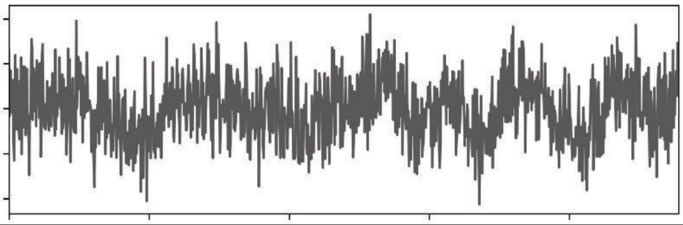	N087_Ch3 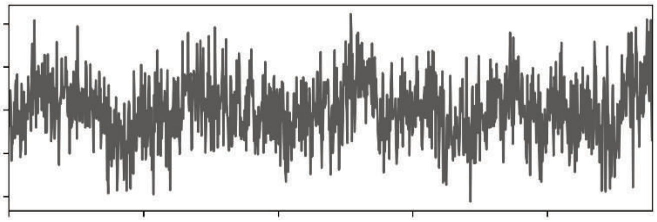
N088_Ch7 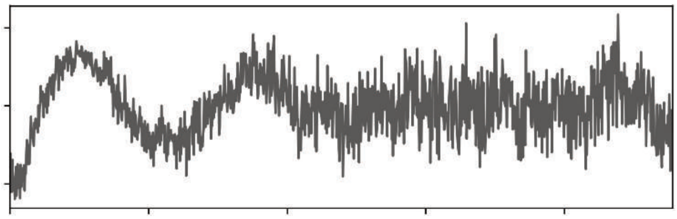	
N089_Ch1 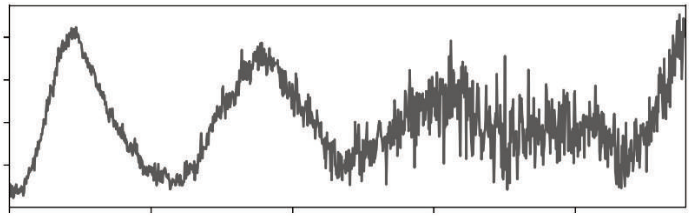	N089_Ch2 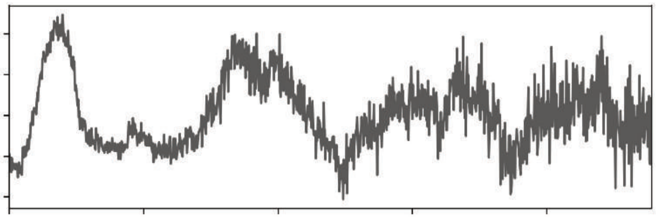

**LBW/ late preterm recordings (n=18)**	
**Replicate 1**	**Replicate 2**
N025_Ch1 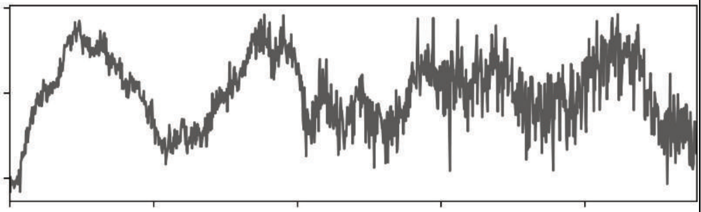	N025_Ch2 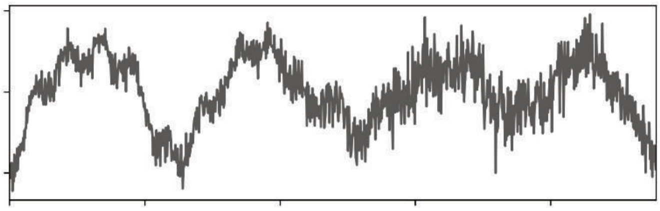
N060_Ch4 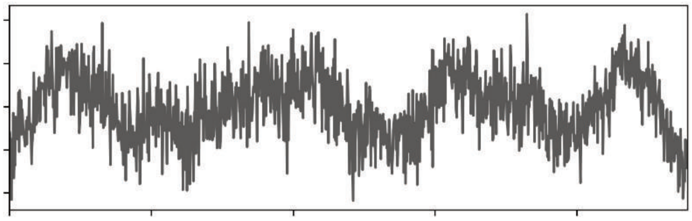	
N064_Ch2 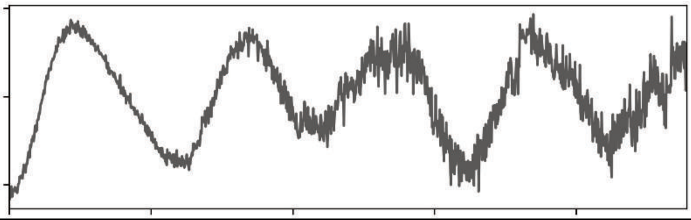	N064_Ch3 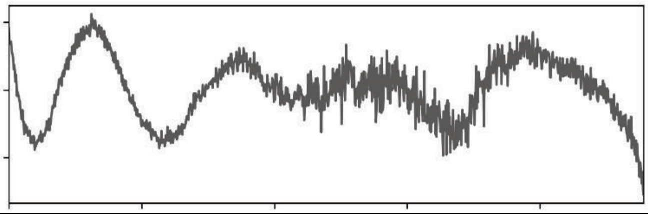
N070_Ch1 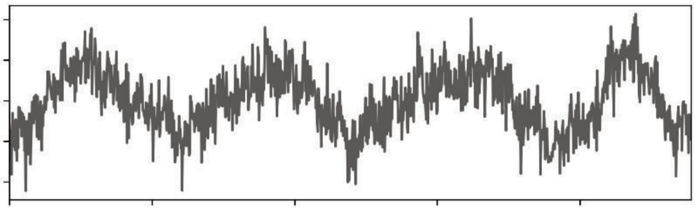	N070_Ch2 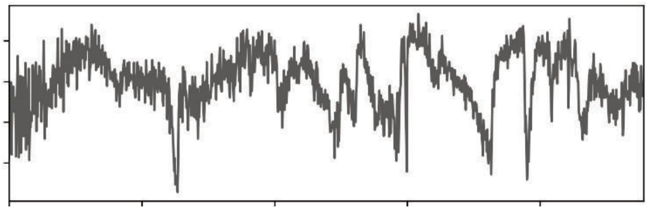
N071_Ch7 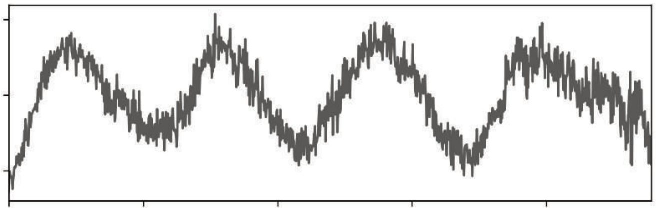	
N074_Ch3 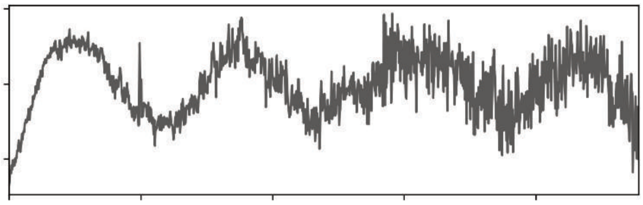	N074_Ch4 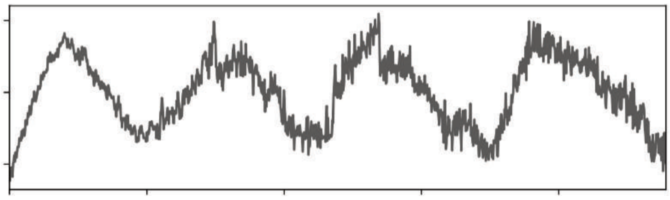

N076_Ch7<br>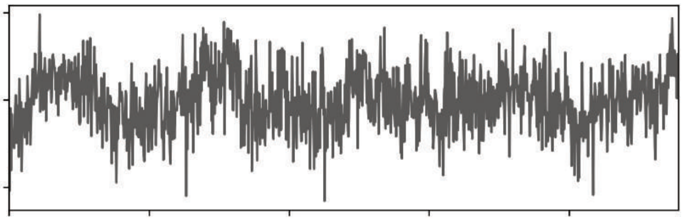	
N080_Ch3<br>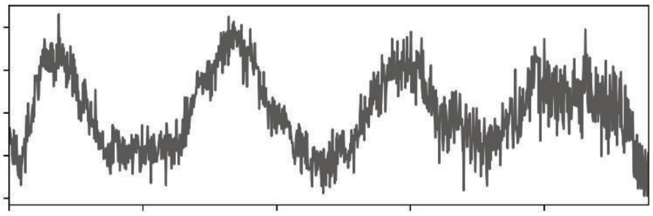	N080_Ch4<br>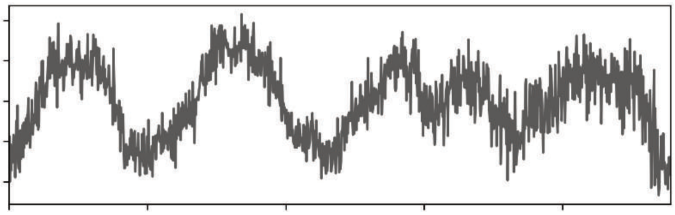
N082_Ch7<br>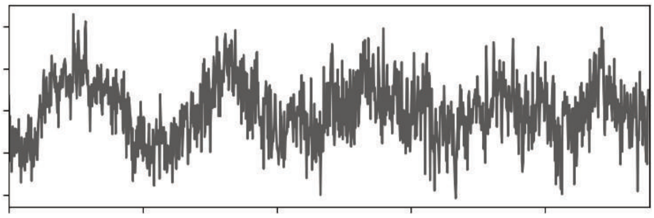	N082_Ch8<br>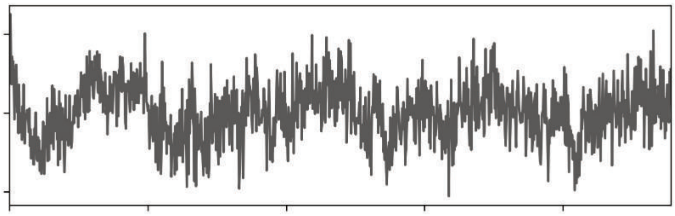
N084_Ch4<br>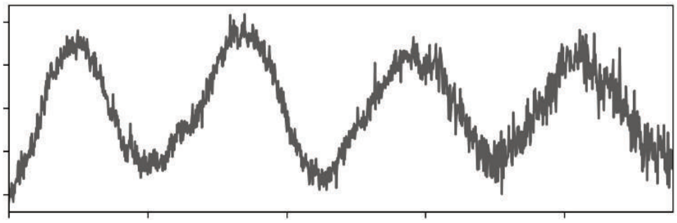	
N085_Ch5<br>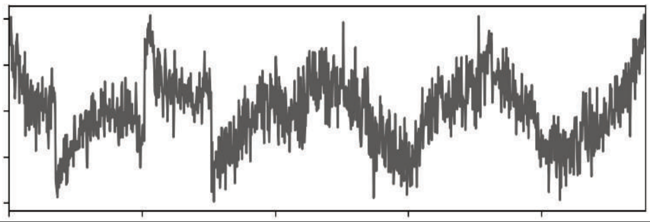	N085_Ch6<br>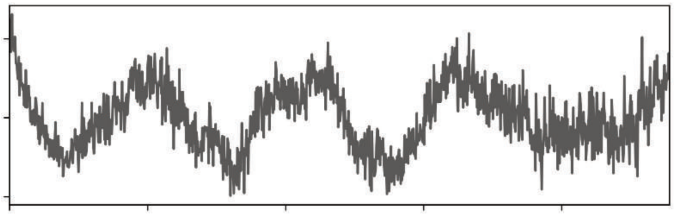
N086_Ch1<br>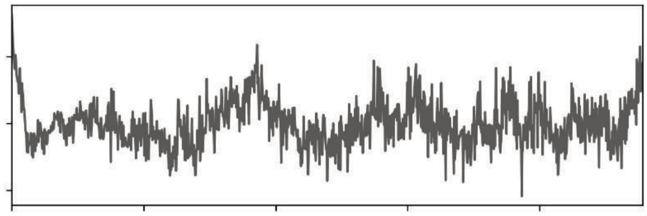	
N092_Ch1<br>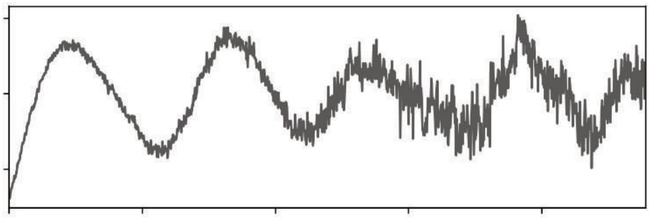	N092_Ch3<br>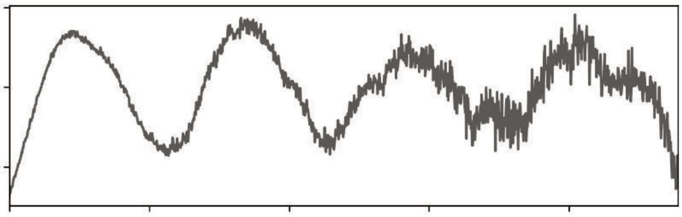

N093_Ch1 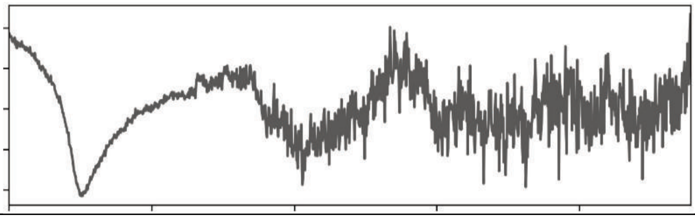	N093_Ch3 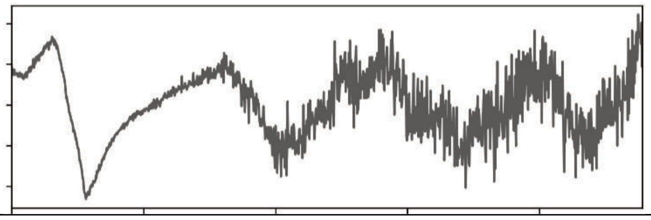
N094_Ch3 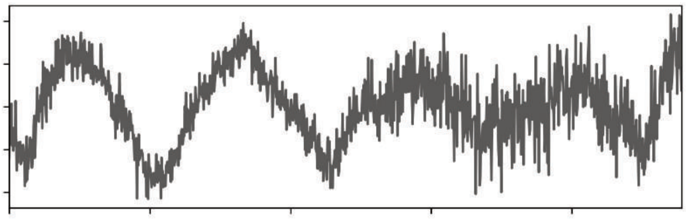	N094_Ch4 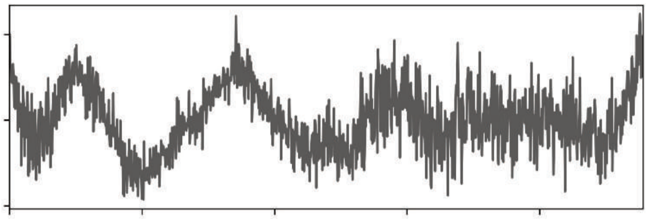
N099_Ch7 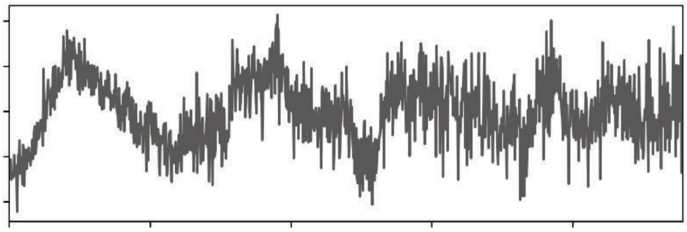	
N100_Ch6 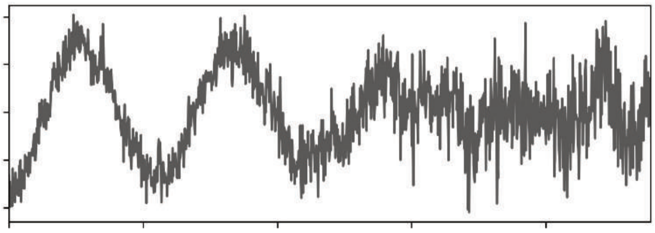	N100_Ch7 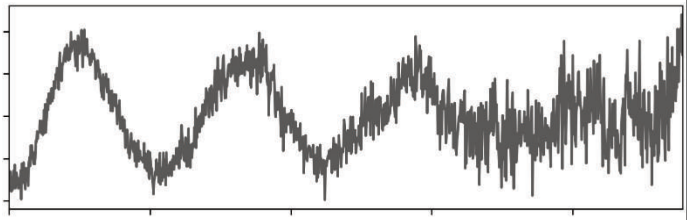
N103_Ch1 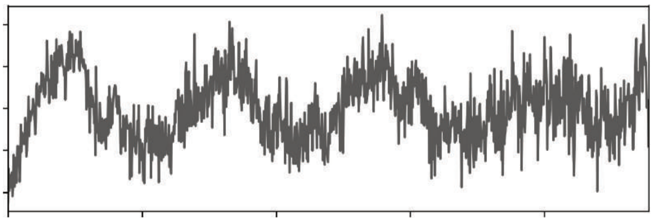	
**VLBW / very preterm recordings (n=7)**	
**Replicate 1**	**Replicate 2**
N036_Ch7 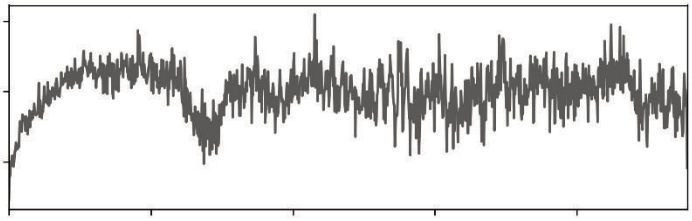	N036_Ch8 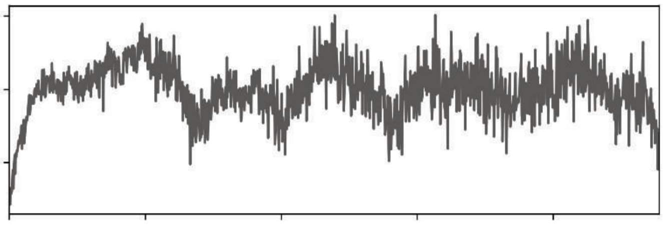

N056_Ch1 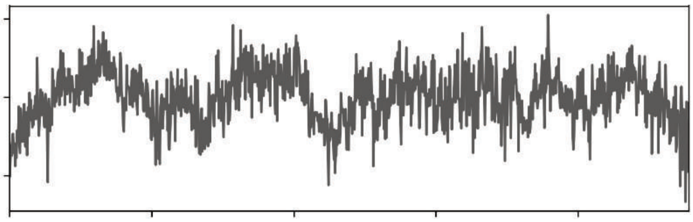	N056_Ch3 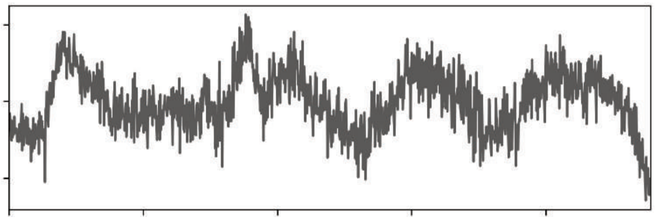
N057_Ch2 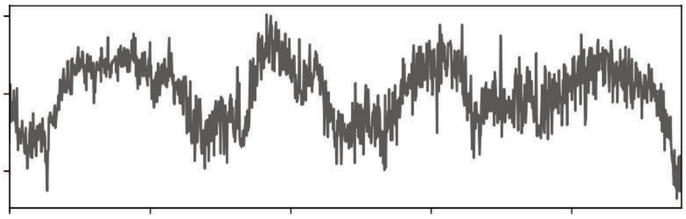	N057_Ch4 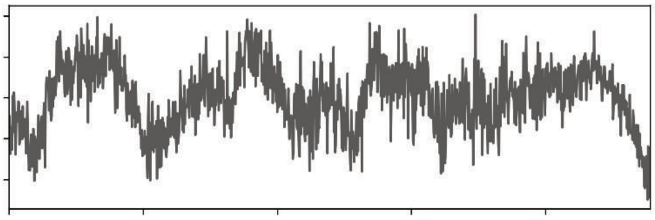
N079_Ch1 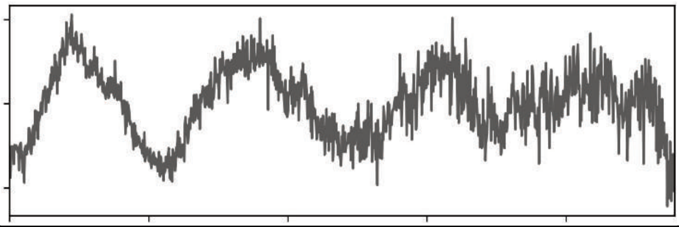	N079_Ch2 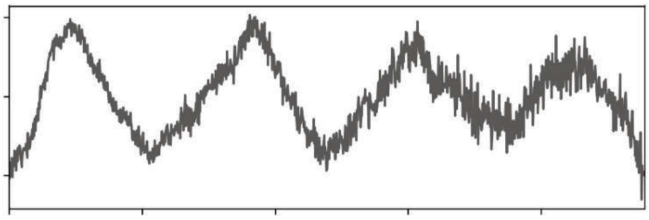
N098_Ch4 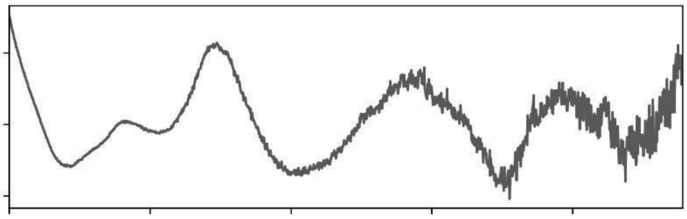	N098_Ch5 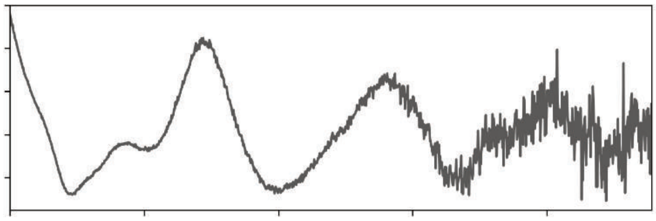
N101_Ch4 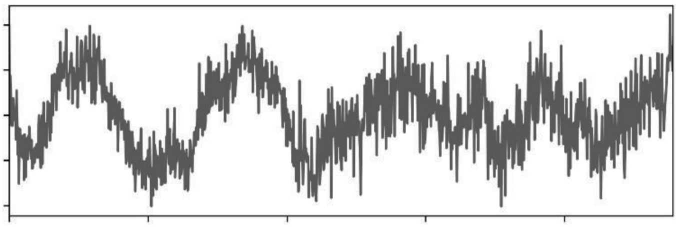	
N102_Ch5 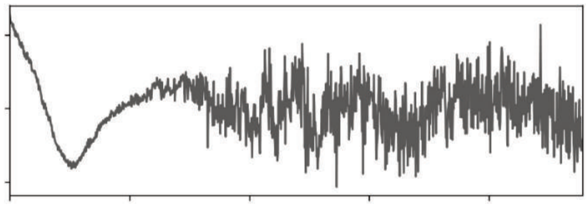	N102_Ch6 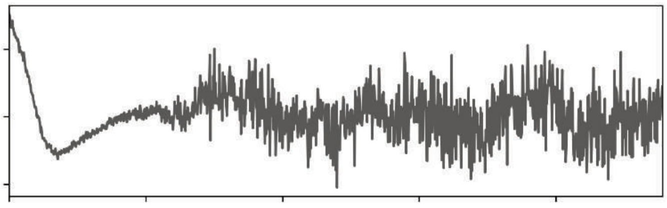

ELBW / extremely preterm recordings (n=9)	
**Replicate 1**	**Replicate 2**
N037_Ch5<br>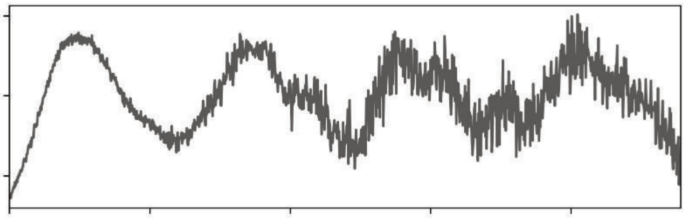	N037_Ch6<br>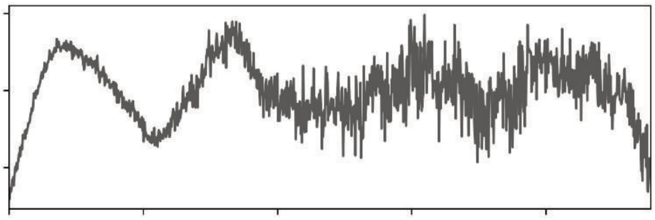
N038_Ch7<br>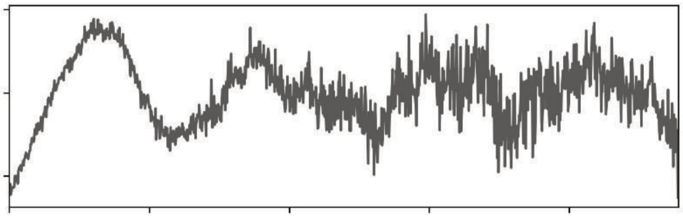	N38_Ch8<br>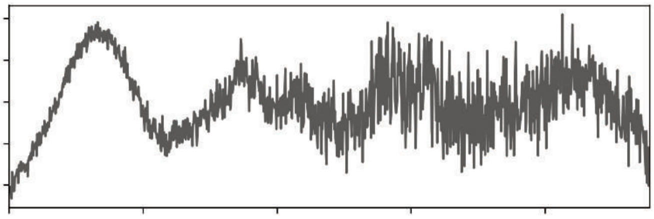
N053_Ch2<br>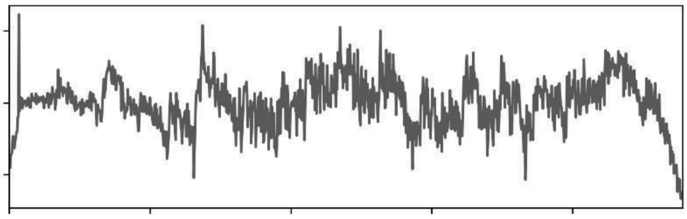	N053_Ch3<br>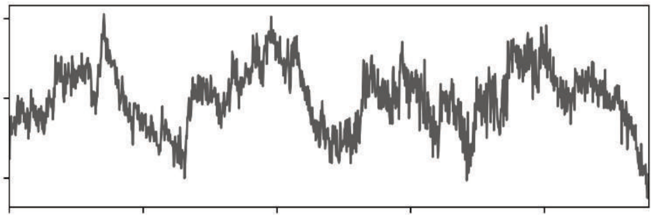
N054_Ch3<br>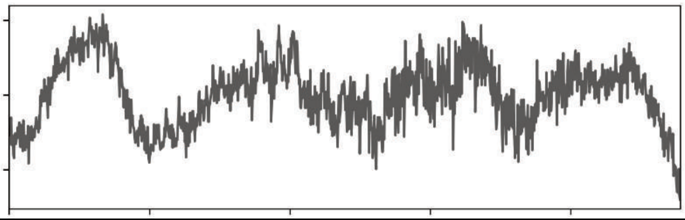	N054_Ch4<br>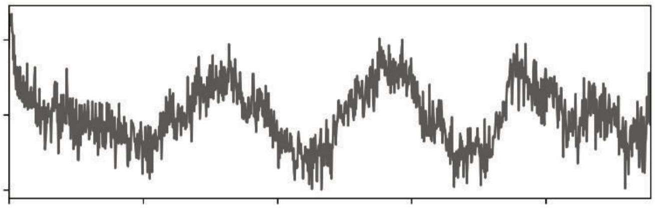
N063_Ch2<br>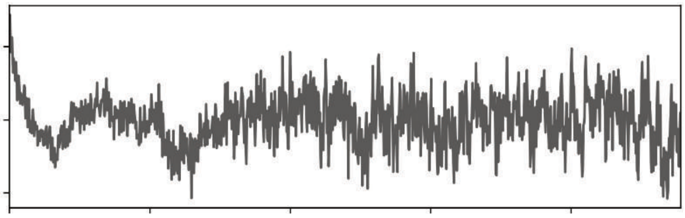	N063_Ch3<br>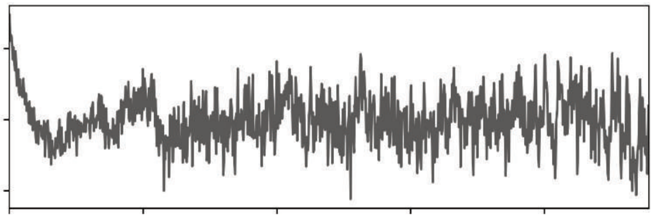
N066_Ch1<br>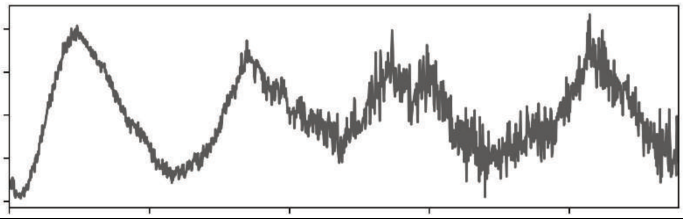	

N068_Ch1 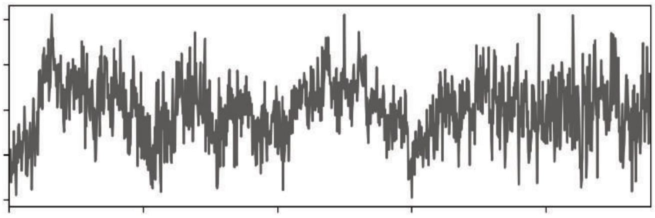	
N083_Ch8 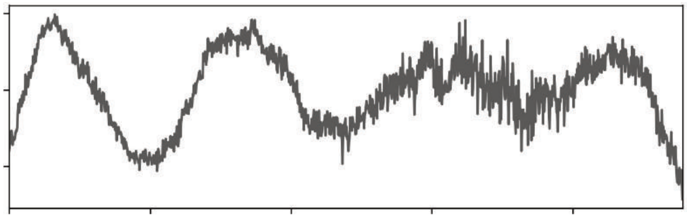	
N095_Ch5 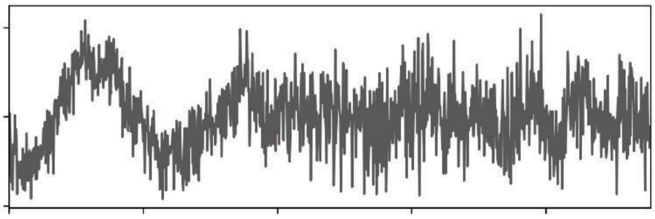	N095_Ch6 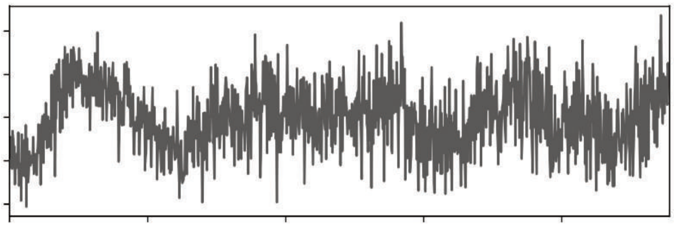

Supplementary Data 3: All HUVEC bioluminescence recordings (12-108 h) included in data analysis, sorted by birth weight classification.

Supplementary Data 4: Correlation results between the circadian parameters within each BW or GA group, respectively.

**BW groups**	**Term**<br>**> 2,500 g** (n=26)<br>**r²; p-value**	**LBW**<br>**2,500 – 1,500 g** (n=18)<br>**r²; p-value**	**VLBW**<br>**1,499 – 1,000 g** (n=7)<br>**r²; p-value**	**ELBW**<br>**< 1,000 g** (n=9)<br>**r²; p-value**
Period vs. Amplitude	ns	ns	ns	0.6527; 0.0084
Period vs. Phase	ns	ns	ns	ns
Period vs. Wavelet power	ns	ns	ns	0.6788; 0.0063
Amplitude vs. Phase	ns	ns	ns	ns
Amplitude vs. Wavelet power	0.8699; <0.0001	0.8868; <0.0001	0.9850; <0.0001	0.9949; <0.0001
Phase vs. Wavelet power	ns	ns	ns	ns
**GA groups**	**Term**<br>**≥ 37+0 wks** (n=23)<br>**r²; p-value**	**Late preterm**<br>**36+6 – 32+0 wks** (n=16)<br>**r²; p-value**	**Very preterm**<br>**31+6 – 28+0 wks** (n=12)<br>**r²; p-value**	**Extremely preterm**<br>**< 28+0 wks** (n=9)<br>**r²; p-value**
Period vs. Amplitude	ns	ns	ns	0.6527; 0.0084
Period vs. Phase	ns	ns	ns	ns
Period vs. Wavelet power	ns	ns	ns	0.6788; 0.0063
Amplitude vs. Phase	ns	ns	ns	ns
Amplitude vs. Wavelet power	0.8707; <0.0001	0.8835; <0.0001	0.9417; <0.0001	0.9949; <0.0001
Phase vs. Wavelet power	ns	ns	ns	ns

Supplementary Data 5: Mean expression of core clock and clock-associated genes in preterm vs. term HUVECs. Mean ± SD expression values of the 18 core clock (top) and clock-associated genes (bottom) normalized to four control genes (*GAPDH, PPIA, HPRT1, PSMB2*) in HUVEC samples from infants with a birth weight > 2,500 g (n=6), 1,500 – 2,500 g (n=4), 1,000 – 1,499 g (n=3) and < 1,000 g (n=4) infants. Cells were synchronized and sampled over a 24-52 h time period, then averaged by pooling of equal RNA amounts from each time point. The mean gene expression was not statistically different between subgroups classified according to birth weight but there were some genes that were lower in expression in at least 2 out of 3 preterm groups (highlighted in gray). Core clock and clock-associated gene expression levels of all preterm groups normalized to the > 2,500 g group's mean expression set to 1. The arrows indicate whether the value was from the lower third (red), middle third (yellow) or higher third (green) of the range of all values.

Name	Accession	>2,500g: Mean gene expression	SD	x-fold >2,500g	2,500-1,500g: Mean gene expression	SD	x-fold >2,500g	1,499-1,000g: Mean gene expression	SD	x-fold >2,500g	<1,000g: Mean gene expression	SD	x-fold >2,500g
CRY1	NM_004075.3	106106	8991	1,00	90835	5836	0,86	94318	9503	0,89	92591	8409	0,87
CLOCK	NM_004898.2	98411	12081	1,00	93115	4508	0,95	100424	1705	1,02	94280	3786	0,96
NR1D2	NM_001145425.1	96387	16733	1,00	84340	3963	0,88	101949	6022	1,06	80179	7696	0,83
NR1D1	NM_021724.3	25263	4163	1,00	19632	877	0,78	25030	3180	0,99	20520	760	0,81
BMAL1	NM_001030272.1	23779	3506	1,00	23141	2286	0,97	25121	2923	1,06	20486	1686	0,86
CRY2	NM_001127457.1	17950	2018	1,00	17097	1473	0,95	19106	1169	1,06	14637	1602	0,82
PER2	NM_022817.2	12216	1811	1,00	10708	2683	0,88	11791	1371	0,97	10125	566	0,83
PER3	NM_016831.1	4676	697	1,00	4087	605	0,87	4362	196	0,93	4309	905	0,92
CSNK1E	NM_152221.2	365054	47888	1,00	340780	24849	0,93	409595	20902	1,12	324067	41715	0,89
CSNK1D	NM_001893.3	350221	30899	1,00	347697	15052	0,99	352728	7429	1,01	327480	27640	0,94
NPAS2	NM_002518.3	250744	28623	1,00	213273	29099	0,85	230298	18007	0,92	201501	18635	0,80
ARNTL2	NM_020183.3	192486	33884	1,00	166764	13277	0,87	189140	19405	0,98	168105	7133	0,87
BHLHE40	NM_003670.2	104412	56753	1,00	70359	23842	0,67	136623	20228	1,31	95146	16927	0,91
RORA	NM_134261.2	54947	11992	1,00	52606	6278	0,96	62631	8859	1,14	49221	4274	0,90
CIPC	NM_033426.2	51604	5376	1,00	49548	4381	0,96	51649	4498	1,00	49328	4399	0,96
NFIL3	NM_005384.2	31334	5471	1,00	29592	1801	0,94	36051	5629	1,15	27745	3089	0,89
CIART	NM_144697.2	6338	1153	1,00	6428	1198	1,01	6777	1622	1,07	5518	742	0,87
DBP	NM_001352.3	4641	1439	1,00	4408	1501	0,95	4626	349	1,00	3162	625	0,68

__Supplementary Data 6__

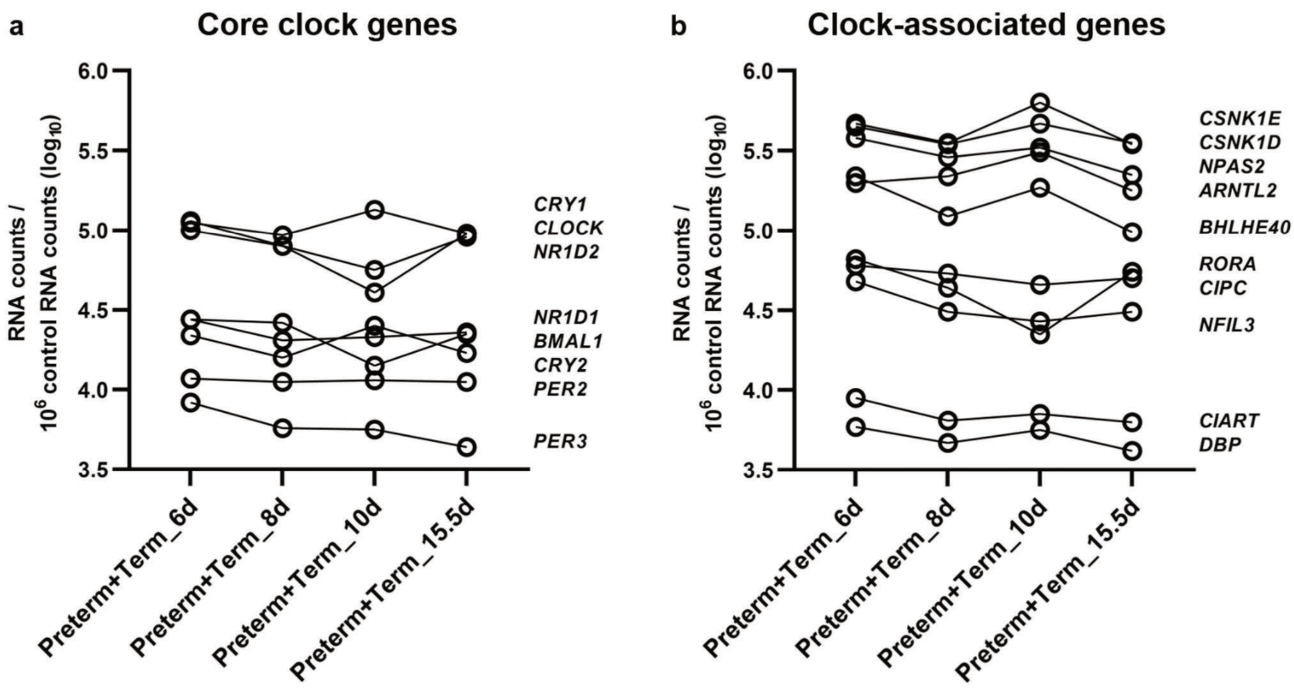

Supplementary Data 6: Mean expression of core clock (a) and clock-associated genes (b) in pooled preterm and term HUVECs, sorted by culture time after isolation. Displayed is the mean expression of 8 core clock and 10 clock-associated genes normalized to four control genes (*GAPDH, PPIA, HPRT1, PSMB2*) in HUVEC samples 6 d (n=5), 8 d (n=5), 10 d (n=4) and 15.5 d (n=17, normal culture time before bioluminescence recording) after isolation. The mean gene expression was not statistically different between the different culture times (one-way ANOVA).
